# Oncofertility information interventions in patients with cancer: A systematic review and meta-analysis

**DOI:** 10.1016/j.apjon.2026.100954

**Published:** 2026-04-13

**Authors:** Mengyan Hao, Yinan Wang, Dandan Zhang, Ying Huang, Huang Tang, Biqing Han, Xuemei Zhong, Huan Li, Ting Liu

**Affiliations:** a School of Nursing, Sun Yat-sen University, Guangzhou, China; b Department of Breast, Guangdong Women and Children Hospital, Guangzhou, China; c Department of Gynecology, Guangdong Women and Children Hospital, Guangzhou, China; d Department of Thyroid and Breast Surgery, The Third Affiliated Hospital, Sun Yat-sen University, Guangzhou, China

**Keywords:** Cancer, Fertility, Oncofertility information interventions, Meta-analysis, Systematic review

## Abstract

**Objective:**

Fertility problems are increasingly recognized as a critical challenge for patients with cancer. They are associated with diverse health-related outcomes. This study aims to systematically synthesize and evaluate the impact of oncofertility information interventions in patients with cancer.

**Methods:**

A comprehensive search was conducted in PubMed, Embase, Scopus, Web of Science, and Cochrane Library from inception to November 21, 2025. Randomized controlled trials using oncofertility information interventions among patients with cancer were included. The risk of bias was evaluated using version 2 of the Cochrane risk-of-bias tool for randomized trials (RoB 2). The certainty of the evidence was evaluated using the GRADE system. Meta-analysis was conducted using R software (version 4.5.1). The study was registered in PROSPERO (CRD42025641728).

**Results:**

A total of 11 articles were included in this systematic review, involving 1046 participants, with 10 articles included in the meta-analysis. The pooled analysis showed that oncofertility information interventions could statistically significantly improve patients' fertility-related knowledge (standardized mean difference [SMD] = 0.37, 95% confidence interval [95% CI] = 0.01, 0.72; moderate quality of evidence), reducing reproductive concerns (mean difference [MD] = −26.34; 95% CI = −27.86, −24.82; moderate quality of evidence), decisional conflict (MD = −5.83, 95% CI = −10.49, −1.17; moderate quality of evidence), and decisional regret (SMD = −0.57, 95% CI = −1.12, −0.02; low quality of evidence). Subgroup analysis indicated that the non-Asian group, multiple cancers group, multi-session group, online interventions group, and hybrid interventions group were more effective for fertility-related knowledge (*P*_s_ < 0.05).

**Conclusions:**

This meta-analysis provides low to moderate quality evidence that oncofertility information interventions were associated with improvements in fertility-related knowledge and reductions in reproductive concerns, decisional conflict, and decisional regret among patients with cancer. Further well-designed and large-scale studies are needed to confirm their effectiveness and to guide broader clinical practice.

## Introduction

Cancer represents a critical public health issue with profound implications for economic and social development worldwide.^1^ The incidence of cancer is increasingly shifting toward younger populations.^2^^,^^3^ Advances in early detection and multimodal treatments have substantially improved patient survival rates,^4^ further contributing to a growing population of patients with cancer of childbearing age. Many of these patients have a strong desire to preserve their fertility and to have biological children.^5^ However, common cancer therapies, such as chemotherapy, radiotherapy, and surgery, can directly lead to the loss of reproductive organs or impair ovarian and uterine function, thereby reducing fertility and often resulting in temporary or permanent infertility.^6^^,^^7^ Moreover, psychological distress following a cancer diagnosis may disrupt hormonal balance and contribute to ovarian dysfunction.^8^^,^^9^ These direct and indirect effects on fertility often give rise to fertility problems among patients with cancer.^10^

Fertility problems persist throughout the entire continuum of cancer diagnosis, treatment, and long-term survivorship, imposing a substantial psychological burden that significantly diminishes patients' quality of life.^11^^,^^12^ Critically, elevated fertility problems are closely associated with increased decisional conflict and regret, as well as compromised quality of decision-making.^13^^,^^14^ These issues can undermine treatment adherence, leading to clinical delays or premature discontinuation of therapy, which adversely affect survival outcomes.^15^ On the other hand, untreated fertility problems disrupt patients’ reproductive decision-making, hinder timely fertility preservation, and ultimately compromise the attainment of reproductive goals.^16^ Addressing these challenges extends beyond psychosocial support and should be recognized as a fundamental component of comprehensive, patient-centered care. Such efforts are crucial for simultaneously optimizing therapeutic outcomes and supporting informed reproductive decision-making.

A variety of interventions have been developed to address fertility problems among patients with cancer, such as mindfulness-based interventions^17^ and intimacy enhancement interventions designed to improve communication.^18^ However, a major limitation of these approaches is that they tend to alleviate reproductive concerns indirectly by improving emotional well-being and social support, without addressing the issue at its core. In contrast, oncofertility information interventions appear to provide a more direct and effective strategy.^12^ The American Society of Clinical Oncology and the European Society for Medical Oncology recommend that fertility-related discussions should be initiated early in the cancer treatment trajectory for patients of reproductive age.^19^^,^^20^

Oncofertility information support refers to the structured and evidence-based delivery of accurate and comprehensible information on fertility risks and preservation options associated with cancer. It aims to enhance treatment adherence and clinical outcomes while facilitating informed reproductive decision-making.^21^^,^^22^ Several qualitative systematic reviews have contributed to a deeper understanding of oncofertility information support among patients with cancer. Effective oncofertility information interventions may improve patients’ fertility-related knowledge, reduce decisional conflict, and facilitate informed choices regarding cancer treatment and fertility preservation.^23^

However, the limited number of studies has precluded quantitative synthesis in previous reviews. Inconsistent effectiveness and uncertainty regarding the optimal components of interventions continue to hinder the development of standardized, evidence-based clinical guidance. Therefore, a systematic review and meta-analysis is warranted to quantitatively synthesize the existing evidence. This study examines the effects of oncofertility information interventions on a broad spectrum of patient-centered health outcomes, such as cognitive, psychological, and decision-making outcomes. The findings may provide comprehensive evidence to inform future intervention development and optimize clinical practice.

## Methods

### Reporting guidelines

A systematic review and meta-analysis were conducted in accordance with the Cochrane Handbook for Systematic Reviews of Interventions.^24^ The results were reported in compliance with the Preferred Reporting Items for Systematic Reviews and Meta-Analyses (PRISMA) 2020 Statement.^25^ The study protocol was registered with PROSPERO (CRD42025641728).

### Search strategy

Two reviewers independently conducted a comprehensive literature search across PubMed, Web of Science, Scopus, Embase, and the Cochrane Library, covering all records from inception to November 21, 2025. The search strategy incorporated Medical Subject Headings terms, keywords, and free words, including but not limited to “neoplasms,” “cancer,” “fertility,” “counseling,” and “randomized controlled trial.” The complete search strategies for each database are provided in Supplementary Table S1. Additionally, the reference lists of all included studies were manually screened to identify further relevant publications. Only studies published in English were eligible for inclusion. All disagreements were resolved by discussion with a third reviewer.

### Study eligibility criteria

In this study, the PICOS framework (Population, Intervention, Comparison, Outcome, and Study design) was employed to define the eligibility criteria for study selection.

#### Population

All patients diagnosed with cancer were eligible, irrespective of age, cancer type, stage, or treatment received.

#### Intervention

This review included any intervention that provided patients with fertility-related information, which is defined as a comprehensive set of knowledge and guidance provided to patients concerning fertility and reproductive health issues associated with cancer and its treatment. This information encompasses key components such as the potential impact of various cancer therapies on fertility, individualized assessment of infertility risk, and available fertility preservation techniques.^26^^,^^27^ Fertility-related information may be disseminated through multiple intervention modalities, such as counseling, paper brochures, decision aids, or video education. Moreover, these interventions could be delivered by any medium (e.g., face-to-face, telephone, or online).

#### Comparison

The study did not restrict the types of control groups. Usual care, a waitlist, or other types of interventions were all eligible.

#### Outcomes

The study included a broad range of outcomes without predefined restrictions, covering multiple domains of patient-centered health outcomes.

#### Study design

The studies included in this meta-analysis were randomized controlled trials. Reviews, protocols, editorials, meta-analyses, conference abstracts, other secondary sources, and letters to the editor were excluded.

### Data selection and extraction

All retrieved records were imported into EndNote X21 to remove duplicates. Subsequently, two independent reviewers screened titles and abstracts to identify studies that potentially met the predefined inclusion criteria. Full-text articles of the selected studies were then assessed for eligibility. Any disagreements between the reviewers were resolved through discussion or consultation with a third reviewer.

Data extraction was conducted independently by two reviewers using a predesigned structured form. Extracted information included publication details (author, year, country), participant characteristics (disease type, age, sex), intervention specifics (components, delivery mode, duration), control group information, follow-up, and outcome measurements at the last follow-up. The extracted data were cross-checked to ensure accuracy. For studies with missing or unclear data, attempts were made to contact the original authors to obtain the necessary information. Studies lacking sufficient outcome data were excluded from the analysis.

### Risk of bias assessment

The quality of the included randomized controlled trials was independently assessed by two reviewers using the Cochrane Risk of Bias 2 (RoB 2) tool.^28^ The tool evaluates five domains: the randomization process, deviations from intended interventions, missing outcome data, measurement of outcomes, and the selection of reported results. Each domain was judged as having a low risk of bias, some concerns, or high risk of bias. Disagreements between the reviewers were resolved through discussion or consultation with a third reviewer.

### Data synthesis and analysis

All statistical analyses were completed using the meta package in R (version 4.5.1). For continuous outcomes, depending on the consistency of measurement tools across studies, effect sizes were reported as either standardized mean difference (SMD) or mean difference (MD), with their corresponding 95% confidence intervals (95% CIs). Specifically, MD was used when the same measurement instrument was applied across studies, whereas SMD was used when different instruments were employed. Statistical heterogeneity among studies was assessed using the *I*^2^ statistic. A value below 50% was considered indicative of low heterogeneity, justifying the use of a fixed-effect model for effect estimation, while a random-effects model was applied otherwise.^29^ Given the potential heterogeneity beyond measurement tools, subgroup analyses were performed, where applicable, based on participant and intervention characteristics to explore sources of heterogeneity. To evaluate the robustness of the findings, leave-one-out sensitivity analyses were conducted by sequentially excluding each included study and recalculating the pooled effect. Publication bias was assessed using funnel plots.

### Certainty of evidence

Evidence quality was evaluated according to the Grading of Recommendation, Assessment, Development, and Evaluation (GRADE) system.^30^ Although randomized controlled trials are initially considered high-quality evidence, they may be downgraded due to limitations such as risk of bias, inconsistency, indirectness, imprecision, or publication bias. All authors independently evaluated the evidence and resolved discrepancies through discussion.

## Results

### Study selection

**Fig. 1:**
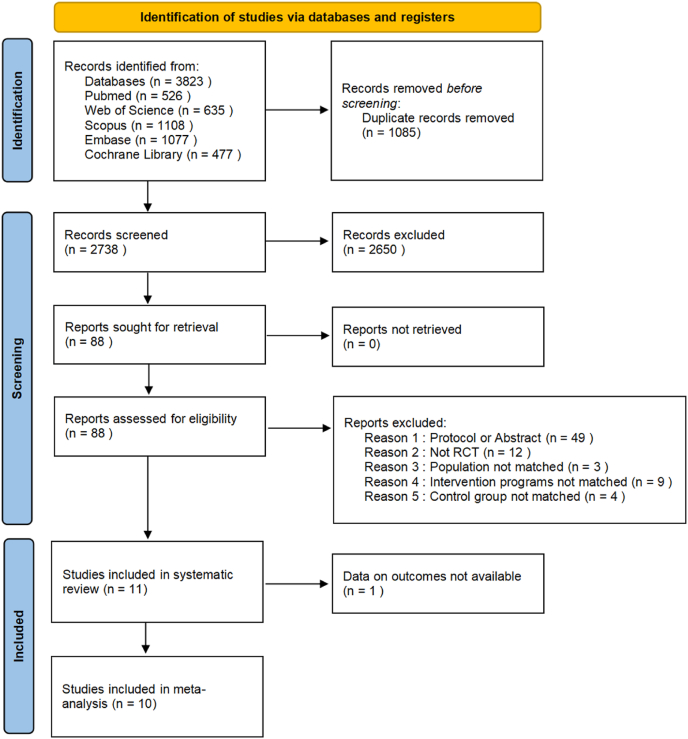
PRISMA flow diagram illustrating the systematic approach to study selection. PRISMA, Preferred Reporting Items for Systematic reviews and Meta-Analyses.

A total of 3823 potentially relevant records were identified through the initial database search. After the removal of 1085 duplicates, 2738 records remained for title and abstract screening. Subsequently, 2650 records were excluded for not meeting the predefined inclusion criteria. The remaining 88 full-text articles were retrieved and assessed for eligibility. As a result, 11 articles were included in the systematic review. Of these, 10 articles representing 9 independent studies provided sufficient outcome data and were thus included in the meta-analysis (Fig. 1).

### Characteristics of included studies

#### Participants

The included articles were published between 2007 and 2025. Three articles were conducted in America, and one each in Iran, France, Japan, Sweden, mainland China, and Taiwan (China). In addition, two articles were from the same multicenter study conducted in Switzerland and Germany. A total of 1046 participants were included across all articles in this review. Among the included articles, one focused on cervical cancer, four on breast cancer, and six on a variety of cancer types. None of the included studies reported any serious adverse events. The detailed characteristics of the included studies are shown in Table 1.

**Table 1:** Characteristics of included studies.

Author, year, country	Participates	Sample size	Intervention group	No treatment group	Outcome (measures)	Data collection point	Retention rate
Barjasteh et al. (2022) Iran	Female patients with breast cancer aged 18–49 at stage I, II or IIIa	IG: 50 CG: 50	Group counseling **Content:** Reproductive and sexual counseling based on “Good Enough Sex”. **Delivery mode:** Offline Face-to-face Grouping (couples) **Provider:** By Researcher **Dosage:** Four sessions of 90–120 min each; 1 month	Waitlist	● Anxiety and depression (DASS-21) ● Reproductive concerns (RCAC)	Baseline 3 months 4 months	97%
Canada et al. (2007) America	Adolescent and young adult patients with cancer aged 15-25	IG: 12 CG: 12	Counseling sessions **Content:** Provide counseling sessions related to fertility, sexual development, etc., and distribute the “workbook.” one month after the counseling sessions, conduct a “booster” phone call. **Delivery mode:** Both online and offline Face-to-face and video call One-to-one **Provider:** By a doctoral level clinical psychologist **Dosage:** Two sessions of 90 min each, plus one additional booster” phone call.	Waitlist	● Fertility-related knowledge (Study-specific) ● Anxiety and depression (BSI 18) ● Quality of life (CARES: Dating subscale)	Baseline Post-intervention^a^ 3 months^b^	79.17%
Dong et al. (2024) China	Patients with cervical cancer aged 20–49 years	IG: 33 CG: 33	Narrative nursing **Content:** Health education related to reproductive and narrative nursing intervention. **Delivery mode:** Both online and offline Face-to-face and phone One-to-one **Provider:** By nursing graduate students **Dosage:** Total five intervention sessions at key moments: Upon admission, the day before surgery, the first day after surgery, the day before discharge, and one week after discharge, with each session lasting no more than 40 min; Admission to 1 week post-discharge	Standard nursing	● Quality of life (FACT-Cx) ● Reproductive concerns (RCAC)	Baseline Post-intervention^a^	95.45%
Ehrbar et al. (2019) (2021) Switzerland and Germany	Newly diagnosed female patients with cancer aged 18 to 40	IG: 40 CG: 39	Online decision aid about fertility preservation **Content:** The decision aid provides comprehensive information on cancer-related fertility risks and fertility preservation procedures, as well as interactive value-clarification exercises. **Delivery mode:** Online **Provider:** By online decision aid **Dosage:** The decision aid can be accessed at any time. Participants accessed the intervention 2.23 times on average, with sessions lasting 9 min 10 sec; 12 months	Usual care	● Decisional conflict (DCS) ● Decisional regret (DRS) ● Fertility intention (Study-specific)	Baseline 1 month 12 months	46.84%
Huang et al. (2022) Taiwan, China	Female patients with breast cancer under the age of 50 who are about to start cancer treatment.	IG: 36 CG: 35	Oncofertility education program **Content:** Naturalistic decision-making model-based oncofertility care education. **Delivery mode:** Offline Face-to-face Continue **Provider:** Nurses educated in oncofertility **Dosage:** Continuous nursing intervention during the initial hospitalization for treatment; during hospitalization	Attention control	● Fertility-related knowledge (IKQ) ● Decisional conflict (DCS) ● Fertility intention (FIS)	Baseline Post-intervention^a^	85.92%
Huyghe et al. (2009) France	Male patients with cancer aged 14 to 45	IG: 10 CG: 10	Banking on Fatherhood (BOF) educational tool **Content:** Educational videos related to fertility **Delivery mode:** Online **Provider:** By professionals **Dosage:** One session of 45–60 minutes	Usual care	● Fertility-related knowledge (CPS) ● Decisional conflict (DCS)	Baseline 45–60 minutes	100%
Koizumi et al. (2023) Japan	Female patients with early-stage breast cancer aged 20–39 years	IG: 37 CG: 37	Oncofertility! Psycho-education and couple Enrichment (O!PEACE) therapy **Content:** Focused on fertility decision-making, stress coping, and couple resilience **Delivery mode:** Offline Face-to-face Grouping (couples) **Provider:** By reproductive psychologists **Dosage:** Two sessions of 70 min each; 2 or more days	Usual care	● Fertility-related knowledge (Study-specific) ● Anxiety and depression (HADS)	Baseline Post-intervention^a^	93.24%
Micaux et al. (2022) Sweden	Diagnosed with a malignant brain, breast, cervical, ovarian, testicular cancer, or lymphoma at age 18–39, approximately 1.5 years prior	IG: 64 CG: 60	Fertility and Sexuality following cancer (Fex-can) intervention **Content:** Web-based, automated self-help psychoeducational program aimed at reducing fertility-related distress and related psychosocial outcomes. **Delivery mode:** Online **Provider:** By network platform **Dosage:** The intervention required participants to spend at least 30 minutes per week on the intervention website; 3 months	Standard nursing	● Fertility-related knowledge (previously developed) ● Anxiety and depression (HADS) ● Quality of life (EORTC-QLQ-C30)	Baseline 3 months 6 months	81.45%
Partridge et al. (2019) America	Patients with breast cancer aged 18 to 45 at stages I to III	IG: 245 CG: 222	Young & Strong Study **Content:** An educational and supportive care program including paper manuals with web-based educational resources. **Delivery mode:** Both online and offline **Provider:** By printed booklet and online platform **Dosage:** The intervention provided educational resources with on-demand access throughout the program; 12 months	Attention control	● Attention to fertility	Baseline 3 months 6 months 12 months	99.57%
Nahata et al. (2025) America	Male patients with cancer aged 12 to 25	IG: 10 CG: 11	Family-centered adolescent Sperm banking values clarification tool (FAST) **Content:** Facilitates sperm banking communication and decision-making through fertility preservation discussions. **Delivery mode:** Both online and offline Face-to-face Grouping (family) **Provider:** By research staff with master’s degree **Dosage:** One session ranged from 4 min 52 s to 14 min 1 s, with a median duration of 10 min 10 s	Standard nursing	● Banking attempts ● Decisional regret (BSDQ)	Baseline 1 month	80.95%

BSDQ, Modified Brief Subjective Decision Quality Measure; BSI 18, Brief Symptom Inventory 18; CARES, Dating Subscale of the Cancer Rehabilitation Evaluation System; CG, Control Group; CPS, Cancer and Parenthood Scale; DASS-21, Depression Anxiety Stress Scales-21; DCS, Decisional Conflict Scale; DRS, Decision Regret Scale; EORTC QLQ-C30, European Organization for Research and Treatment of Cancer Quality of Life Questionnaire-Core 30; FACT-Cx, Functional Assessment of Cancer Therapy-Cervix; FIS, Fertility Intention Scale; HADS, Hospital Anxiety and Depression Scale; IG, Intervention Group; IKQ, Infertility Knowledge Questionnaire; RCAC, Reproductive Concerns After Cancer Scale.

∗The retention rate was calculated based on data from the last follow-up.

a Total duration of intervention not reported.

b Post-intervention.

#### Interventions

Table 1 summarizes the intervention characteristics of the included studies. The interventions were categorized by delivery mode as offline, online, and hybrid. Offline interventions involved face-to-face counseling,^31^ psychological education,^32^ and oncofertility nursing.^33^ Online interventions included decision aids,^34^^,^^35^ fertility-related videos,^36^ and web-based psychoeducation.^37^ Hybrid interventions combined elements of both formats, such as offline counseling with online augmentation calls,^38^ offline fertility care instruction with offline–online combination of narrative care,^39^ tailored discussions conducted either face-to-face or via videoconferencing/phone,^31^^,^^33^^,^^36^, ^37^, ^38^, ^39^, ^40^ and paper manuals with web-based educational resources.^41^

The frequency and dosage of interventions varied across studies. Specifically, five articles reported both components, with the number of sessions ranging from one to five and each session lasting less than 120 minutes. The remaining articles described the intervention procedures, but due to their ongoing nature, no specific schedule was provided. Additionally, the reported retention rates ranged from 46.84% to 100%.

#### Control groups

The articles employed various control conditions, including usual care, waitlist, standard nursing, and attention control.

#### Outcome measures

The outcomes reported in the included studies spanned several domains of patient-centered health outcomes, including cognitive, psychological, decision-making, and quality of life dimensions. Cognitive outcomes primarily referred to fertility-related knowledge, reflecting patients' understanding of fertility risks and available preservation options associated with cancer treatment. Psychological outcomes included reproductive concerns as well as anxiety and depression, which capture patients' emotional responses to fertility uncertainty and the potential impact of cancer on future reproductive plans. Decision-making outcomes comprised decisional conflict and decisional regret, representing patients' experiences during the process of making fertility-related decisions and their subsequent evaluations of those decisions. Quality of life outcomes reflected the broader influence of cancer and fertility-related issues on patients’ overall well-being. Collectively, these outcomes provide a comprehensive perspective on the potential effects of oncofertility information interventions and were therefore included in this review.

Five articles assessed fertility-related knowledge using the Infertility Knowledge Questionnaire, the Cancer and Parenthood Scale, previously developed instruments, and study-specific questionnaires. Two articles examined reproductive concerns using the Reproductive Concerns After Cancer Scale. Four articles assessed anxiety and depression using the Depression Anxiety Stress Scales–21, the Brief Symptom Inventory 18, and the Hospital Anxiety and Depression Scale. Three articles evaluated quality of life using the Functional Assessment of Cancer Therapy–Cervix, the European Organization for Research and Treatment of Cancer Quality of Life Questionnaire–Core 30, and the Dating Subscale of the Cancer Rehabilitation Evaluation System. Three articles examined decisional conflict using the Decisional Conflict Scale. Two articles assessed fertility intention using the Fertility Intention Scale and study-specific questionnaires. Two articles assessed decisional regret using the Decision Regret Scale and the Modified Brief Subjective Decision Quality Measure.

### Risk of bias assessment

**Fig. 2:**
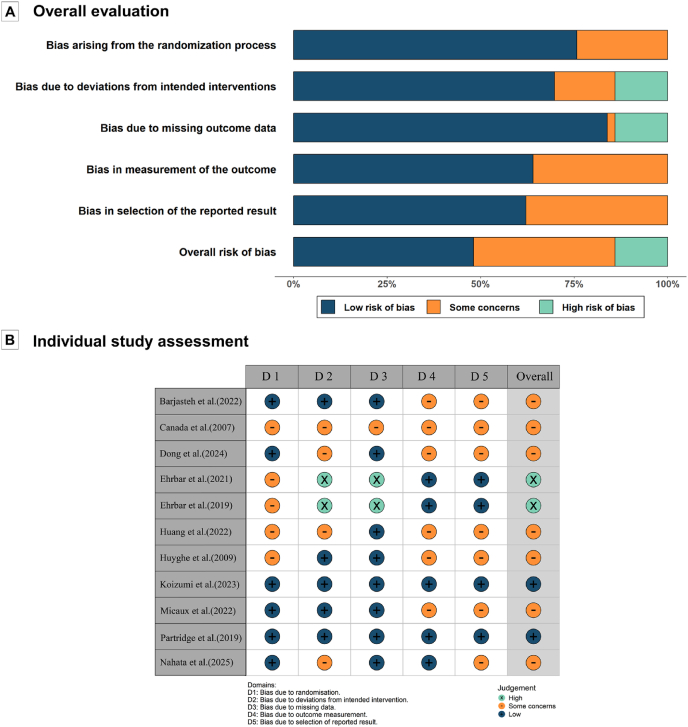
Risk of bias of included studies.

The risk of bias assessments for the included studies are presented in Fig. 2, with detailed results provided in Supplementary Table S2. Among the 11 articles, two were assessed as having a low risk of bias,^32^^,^^41^ two as having a high risk,^34^^,^^35^ and the remaining seven were classified as having some concerns.^31^^,^^33^^,^^36^, ^37^, ^38^, ^39^, ^40^

Regarding the randomization process, five articles were rated as having some concerns due to insufficient information on randomization, while the others were considered low risk. For deviations from intended interventions, two articles were classified as high risk, primarily due to insufficient information regarding deviations, and four articles were assessed as having some concerns due to inappropriate statistical analysis methods. With respect to missing outcome data, one article was rated as having some concerns owing to substantial loss to follow-up, while two articles were classified as high risk because they failed to provide reasons for the high attrition rate. For measurement of the outcome, blinding of outcome assessors was mostly infeasible due to the nature of the outcomes. Furthermore, seven articles were rated as having some concerns because of the absence of prespecified protocols or incomplete statistical analysis plans in the selection of reported results.

### Overall analysis of the effects of oncofertility information support on various health-related outcomes

#### Fertility-related knowledge

**Fig. 3:**
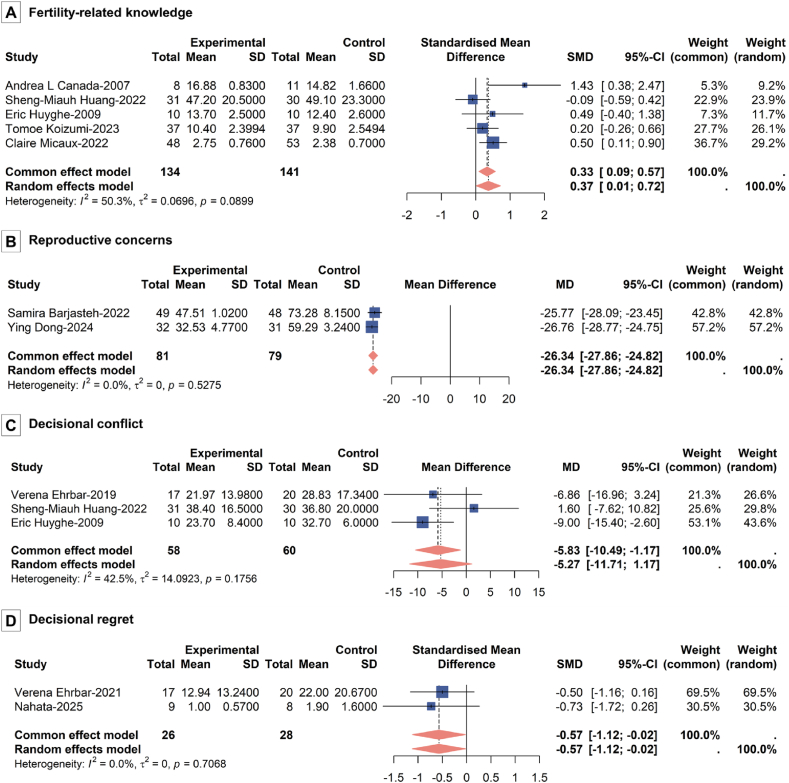
Effect of oncofertility information support on: (A) Fertility-related knowledge, (B) Reproductive concerns, (C) Decisional conflict, and (D) Decisional regret.

A total of five studies evaluated the effects of interventions on fertility-related knowledge. A meta-analysis indicated that oncofertility information support statistically significantly improved patients’ fertility knowledge (Fig. 3A; SMD = 0.37, 95% CI = 0.01, 0.72). These studies exhibited moderate heterogeneity, though the between-study variation was not statistically significant (*P* = 0.09, *I*^2^ = 50.30%).

Subgroup analyses were conducted according to patient and intervention characteristics, where applicable (Table 2, Supplementary Fig. S1).

**Table 2:** The subgroup analysis of the effects of oncofertility information support on fertility-related knowledge.

Categorical moderators	K	SMD	95% CI	*I*^2^ (%)	Between groups	
					Q_b_ (df)	*p*_b_
**Regions**						
Asia	2	0.07	−0.27, 0.41	0.0	4.42 (1)	0.04
Non-Asian	3	0.60	0.26, 0.94	25.9		
**Cancer types**						
Multiple cancers	3	0.60	0.26, 0.94	25.9	4.42 (1)	0.04
Only breast cancer	2	0.07	−0.27, 0.41	0.0		
**Intervention formats**						
Couple	1	0.20	−0.26, 0.66	_	0.56 (1)	0.45
Single	4	0.47	−0.06, 0.99	60.5		
**Delivery modes**						
Hybrid	1	1.43	0.38, 2.47	_	7.37 (2)	0.03
Offline	2	0.07	−0.27, 0.41	0.0		
Online	2	0.50	0.14, 0.86	0.0		
**Session formats**						
Multi-session	3	0.53	0.04, 1.02	56.4	1.59 (1)	0.21
Single-session	2	0.05	−0.39, 0.49	17.1		

CI, Confidence Interval; df, degree of freedom; *p*_b_, *p* between groups; Q_b_, Q between groups; SMD, standardized mean difference.

##### Patient characteristics

Subgroup analyses were conducted to explore the effects of oncofertility information support on fertility-related knowledge across regions and cancer types. Statistically significant differences were observed in both subgroup analyses.

When stratified by region, a statistically significant effect was found in non-Asian populations (SMD = 0.60, 95% CI = 0.26, 0.94). Subgroup analysis showed that interventions for multiple cancers had a statistically significant impact on fertility-related knowledge (SMD = 0.60, 95% CI = 0.26, 0.94).

##### Intervention characteristics

Subgroup analyses by intervention formats, delivery modes, and session formats were undertaken to evaluate the effects of oncofertility information support on fertility-related knowledge. Statistically significant between-group differences were observed for delivery modes and session formats.

For intervention formats, neither single-based interventions (SMD = 0.47, 95% CI = −0.06, 0.99) nor couple-based interventions (SMD = 0.20, 95% CI = −0.26, 0.66) showed statistically significant effects. Regarding delivery modes, both online and hybrid interventions effectively increased fertility-related knowledge (online: SMD = 0.50, 95% CI = 0.14, 0.86; hybrid: SMD = 1.43, 95% CI = 0.38, 2.47).

Furthermore, session formats have opposite effects on fertility-related knowledge. Based on previous studies, the session formats can be classed as multi-session and single-session.^42^ Subgroup analysis showed that multi-session (session number > 1) had a statistically significant impact on fertility-related knowledge (SMD = 0.53, 95% CI = 0.04, 1.02), while single-session (session number ≤ 1) had no statistically significant effect on fertility knowledge (SMD = 0.05, 95% CI = −0.39, 0.49).

#### Reproductive concerns

Two studies that explored reproductive concerns were included in this meta-analysis. The results suggested that oncofertility information support could statistically significantly reduce patients’ reproductive concerns (Fig. 3B; MD = −26.34; 95% CI = −27.86, −24.82). No significant heterogeneity was observed for this outcome (*P* = 0.53, *I*^*2*^ = 0.00%).

#### Decisional conflict

Three studies assessed decisional conflict, and the pooled analysis indicated that oncofertility information support was associated with a statistically significant reduction in decisional conflict (Fig. 3C; MD = −5.83, 95% CI = −10.49, −1.17). Heterogeneity across these studies was moderate but acceptable (*P* = 0.18, *I*^2^ = 42.50%).

#### Decisional regret

Evidence from two studies on decisional regret was included in this meta-analysis. The pooled results suggested that oncofertility information support was associated with reductions in patients’ decisional regret (Fig. 3D; SMD = −0.57; 95% CI = −1.12, −0.02). There was no indication of heterogeneity for this outcome (*P* = 0.71, *I*^*2*^ = 0.00%).

#### Other outcomes

No statistically significant results were found between groups on other outcomes, including quality of life (SMD = 0.43, 95% CI = −1.37, 2.23), anxiety and depression (SMD = −0.90, 95% CI = −2.47, 0.66), and fertility intentions (SMD = 0.10, 95% CI = −0.30, 0.50) (Supplementary Fig. S2).

### Sensitivity analysis

Sensitivity analyses showed effect estimates that were consistent with the direction of the main findings; however, statistical significance varied across scenarios, except for reproductive concerns. This variability suggests that the robustness of the primary results may be limited, and the pooled estimates should therefore be interpreted with caution (Supplementary Fig. S3).

### Publication bias

Due to the limited number of included studies, publication bias was not formally assessed. Funnel plots and statistical tests such as Egger’s test are generally considered unreliable with small numbers of studies. Therefore, while no formal evaluation was performed, the potential for publication bias cannot be completely excluded.

### Certainty of evidence

The certainty of the evidence regarding the effects of oncofertility information interventions on the health-related outcomes of patients with cancer is presented in Supplementary Fig. S4. The quality of evidence was rated moderate for fertility-related knowledge, reproductive concerns, decisional conflict, anxiety and depression, but low for the remaining outcomes. Downgrading was primarily due to concerns about risk of bias, inconsistency, and imprecision.

## Discussion

To the best of our knowledge, this systematic review and meta-analysis is the first to comprehensively evaluate the effects of oncofertility information interventions among patients with cancer. A total of 11 randomized controlled trials involving 1046 participants across various cancer types were included. The pooled results suggested that, compared to control groups, oncofertility information interventions were associated with improvements in patients’ fertility-related knowledge and reduced reproductive concerns, decisional conflict, and decisional regret. These findings are consistent with those of previous systematic reviews.^43^, ^44^, ^45^ Furthermore, subgroup analyses indicated that interventions conducted in non-Asian populations, targeting multiple cancers, providing hybrid or online modes, and implemented with multi-session were associated with statistically significant improvements in fertility-related knowledge, while the effects of intervention formats remained uncertain. The certainty of evidence ranged from low to moderate according to the GRADE assessment, indicating the results should be interpreted with caution. Nevertheless, this review provides preliminary evidence suggesting benefits of oncofertility information interventions for patients with cancer.

Consistent with previous studies,^14^^,^^43^^,^^45^ our research suggests that oncofertility information interventions were associated with improvements in patients' fertility-related knowledge and reduced both decisional conflict and decisional regret. Patients with cancer often face substantial informational gaps regarding fertility risks and preservation options.^46^ Insufficient knowledge is a major contributor to uncertainty and hesitation when making complex treatment decisions.^47^ Limited health literacy and mismatched communication with clinicians further exacerbate these difficulties, hindering effective decision-making and increasing the likelihood of decisional regret following choices made at the time of diagnosis.^48^ Information interventions address these deficits by conveying complex medical information in a systematic and individualized manner.^49^ Such approaches include web-based psychoeducation, face-to-face counseling, and decision aids, all tailored to meet patients' specific needs, thereby enhancing patients' comprehension and retention of knowledge. Improved knowledge, in turn, strengthens patients' ability to evaluate available options and to make choices aligned with their personal values, ultimately reducing decisional conflict and decisional regret.^50^ The observed benefits are consistent with the Ottawa Decision Support Framework, which highlights insufficient knowledge and value uncertainty as key determinants of decisional conflict and decisional regret.^47^ Addressing these gaps through structured interventions can improve patients’ understanding, clarify their values, and foster better decision-making. Future research should explore the long-term effects of these interventions, including how well patients retain and apply the knowledge over time.

Subgroup analyses were conducted to explore potential moderators of intervention efficacy on fertility-related knowledge, though the limited number of studies necessitates cautious interpretation. Interventions conducted in non-Asian populations were more effective than those in Asian settings. Cultural differences in communication styles, social taboos surrounding fertility discussions, and disparities in health literacy may partly explain these differences.^51^ Furthermore, interventions targeting multiple cancers yielded better outcomes than those focused solely on breast cancer. A possible explanation is that broader programs address more diverse fertility-related needs.

In addition, online and hybrid interventions led to significant improvements in fertility-related knowledge, likely due to flexible access, repeated exposure, multimedia support, and the privacy they offer for sensitive topics.^52^^,^^53^ Furthermore, multi-session interventions were more effective than single-session interventions, as repeated exposure helps patients comprehend and retain complex fertility-related information.^54^^,^^55^ These findings suggest that the number of sessions has an important impact on intervention effectiveness.

Taken together, online or hybrid, multi-session interventions may improve knowledge acquisition and retention through repeated exposure, and integrating such technology-supported interventions into routine oncology care could provide more continuous information. Interventions targeting diverse cancer populations may better address heterogeneous fertility-related needs. In Asian settings, culturally sensitive communication and tailored education, supported by training to enhance health care professionals’ cultural competence, should be incorporated.

This study suggests that oncofertility information interventions are associated with reduced reproductive concerns among patients with cancer, which is consistent with previous systematic review.^44^ By providing accurate and timely fertility information, these interventions enhance patients' sense of predictability and control. Such strategies support patients in managing their reproductive health and promote their participation in fertility preservation, thereby reducing uncertainty about their reproductive future and alleviating patients’ worries.^23^ However, some studies indicate that survivors who received fertility counseling reported moderate to high levels of reproductive concerns compared with those who did not.^56^^,^^57^ This discrepancy may be attributed to the substantial risks of infertility and pregnancy-related complications introduced by cancer treatments.^7^^,^^58^ When patients receive comprehensive fertility counseling, they gain a clearer understanding of the potential adverse effects of these treatments on their reproductive capacity, which in turn increases the likelihood that they will report a greater number of reproductive concerns. In addition, for cancer survivors, the reproductive needs vary at different treatment stages; fertility counseling provided during treatment may not be sufficient to address reproductive issues that arise in the post-treatment phase.^10^ Such evidence suggests that reproductive concerns are complex and dynamic. Future interventions should provide sufficient, balanced, and clear information to avoid excessive concerns resulting from incomplete or fragmented counseling. In addition, information provision should be accompanied by training in coping strategies, such as emotional regulation, values clarification, and decision-making support. Moreover, stage-specific and longitudinal counseling services are needed, with careful consideration of the temporal dimension of intervention effects and a clear distinction between short-term and long-term outcomes.

This meta-analysis suggests that oncofertility information interventions did not demonstrate statistically significant improvements in anxiety or depression among patients with cancer. However, previous studies have shown that nurse-led educational interventions can effectively reduce patients' anxiety and depression.^59^ A possible explanation is that prior interventions employed broader educational programs addressing treatment, prognosis, and psychosocial adjustment, thereby exerting a more comprehensive impact on psychological well-being. By contrast, the interventions included in this review primarily focused on fertility-related information, providing relatively narrow content. However, anxiety and depression are multifaceted psychological conditions shaped by diverse factors such as disease severity, treatment outcomes, and social support.^60^ Consequently, interventions targeting only fertility information may be insufficient to address the full spectrum of psychological distress in cancer populations.^61^ These findings emphasize the value of integrative approaches that combine fertility-related support with wider psychosocial care to better meet patients’ psychological needs. Future research should consider multidisciplinary strategies that align reproductive health counseling with interventions targeting broader mental health outcomes in order to achieve more meaningful improvements in anxiety and depression among individuals with cancer.

This study provides preliminary evidence that oncofertility information interventions did not statistically significantly improve quality of life among patients with cancer. However, previous psychoeducational interventions targeting cancer survivors have shown beneficial effects.^62^ Quality of life is a multidimensional construct encompassing physical, psychological, social, and functional domains.^63^ Previous studies employed more comprehensive psychosocial interventions, which have demonstrated greater effectiveness in enhancing overall quality of life. By contrast, interventions focusing solely on reproductive information may be insufficient to yield substantial improvements across these domains.

Furthermore, no statistically significant effect of oncofertility information interventions on patients' fertility intentions was observed in this study. However, the interpretation of this finding is limited due to the small number of included studies and the scarcity of prior research directly addressing this outcome. Consequently, additional high-quality studies, including randomized controlled trials with sufficient sample sizes and standardized outcome measures, are warranted to clarify the impact of fertility information interventions on patients’ reproductive intentions.

This study provides preliminary evidence that oncofertility information interventions were associated with enhanced patients’ fertility-related knowledge while reducing reproductive concerns, decisional conflict, and decisional regret. Nevertheless, several included trials exhibited methodological limitations, including high risk of bias, inconsistency, and imprecision, resulting in low to moderate certainty according to the GRADE framework and uncertainty in the pooled estimates; these limitations may reduce confidence in the estimated effects. However, from a clinical perspective, these interventions remain highly pragmatic. As non-invasive strategies with minimal risk of harm, they may offer potential benefits in improving multiple patient-centered outcomes despite the uncertainty of the current evidence base. Given that some findings are based on a limited number of studies, the results should be interpreted with caution. Future research must prioritize rigorously designed trials with standardized outcome measurements to translate these findings into definitive clinical guidelines.

## Strength and limitations

This review employed a systematic and methodologically rigorous approach to study identification, selection, and evidence synthesis, which helps reduce selection bias and ensures comprehensive coverage of the available trials. As the first meta-analysis to focus exclusively on randomized controlled trials of oncofertility information interventions, it provides more precise effect estimates than previous narrative reviews and offers a clearer understanding of intervention benefits. In addition, the use of sensitivity and subgroup analyses enhances the credibility of the findings by probing potential sources of heterogeneity and clarifying the conditions under which these interventions may be most effective.

Nonetheless, several limitations should be acknowledged in this review. First, the certainty of evidence reflects overall methodological limitations in the included studies, including both the limited number of studies and concerns about study quality. These limitations increase the uncertainty of the pooled estimates, preclude formal assessment of publication bias, and raise concerns about the robustness and generalizability of the findings. Second, this review was restricted to studies published in English, which may have introduced language bias. Taken together, these findings from meta-analysis and subgroup analysis should be considered exploratory and interpreted with caution. Finally, insufficient reporting of comparisons among intervention strategies restricted further exploration of the most effective components. Future research should employ larger sample sizes, adopt standardized intervention protocols, and provide detailed descriptions of intervention characteristics to allow for more precise comparisons and robust conclusions.

## Implications for clinical practice and research

This review provides preliminary evidence that oncofertility information interventions were associated with enhanced fertility-related knowledge and reduced reproductive concerns, decisional conflict, and decisional regret among patients with cancer. Online and hybrid interventions are more effective than offline interventions, and multi-session interventions outperform single-session interventions. Positive effects were also observed in non-Asian populations and in cancer types beyond only breast cancer. In clinical practice, integrating fertility counseling into routine cancer care is essential for providing patients with timely and individualized support. Nurses occupy an advantageous position to provide such support, given their close and continuous contact with patients. Strengthening collaboration with multidisciplinary professionals, particularly those with expertise in fertility care or psychological support, could facilitate the development of more comprehensive and patient-centered guidance in clinical practice. In addition, digital delivery methods can broaden access and facilitate repeated reinforcement, serving as a valuable complement to traditional counseling. To achieve this, coordinated care pathways should be established to ensure consistent and high-quality fertility support. Incorporating fertility support into survivorship planning may further address patients’ long-term reproductive needs and ultimately improve their overall quality of life.

Nevertheless, future research should prioritize rigorous design and comprehensive consideration trials across diverse regions, intervention formats, and cancer types, with standardized outcome measures and longer follow-up to strengthen the evidence base.

## Conclusions

This meta-analysis demonstrated that oncofertility information interventions may improve fertility-related knowledge, reducing reproductive concerns, decisional conflict, and decisional regret among patients with cancer. However, the overall quality of evidence was low to moderate, warranting cautious interpretation of the findings. Future research should employ rigorous designs, provide comprehensive descriptions of intervention content and implementation, and include direct comparisons of tailored interventions to identify the most effective strategies.

## CRediT authorship contribution statement

**Mengyan Hao:** Validation, Methodology, Formal analysis, Data curation, Conceptualization, Writing – review & editing, Writing – original draft. **Yinan Wang:** Validation, Methodology, Formal analysis, Data curation, Writing – original draft. **Dandan Zhang:** Methodology, Investigation, Conceptualization, Writing – review & editing. **Ying Huang:** Validation, Formal analysis, Conceptualization, Writing – review & editing. **Huang Tang:** Validation, Conceptualization, Writing – review & editing. **Biqing Han:** Visualization, Conceptualization, Writing – review & editing. **Xuemei Zhong:** Validation, Conceptualization, Writing – review & editing. **Huan Li:** Conceptualization, Supervision, Project administration, Funding acquisition, Writing – review & editing. **Ting Liu:** Conceptualization, Supervision, Project administration, Funding acquisition, Writing – review & editing. All authors have read and approved the final manuscript.

## Ethics statement

Not required.

## Data availability statement

The authors confirm that the data supporting the findings of this study are available within the article and its supplementary materials.

## Declaration of generative AI and AI-assisted technologies in the writing process

No AI tools/services were used during the preparation of this work.

## Funding

This work was supported by the Guangdong Planning Office of Philosophy and Social Science (Grant No. GD24YSH07); and the Guangzhou Concord Medical Humanities Research and Education Fund (Grant No. 23000-3050070). The funders had no role in considering the study design or in the collection, analysis, interpretation of data, writing of the report, or decision to submit the article for publication.

## Declaration of competing interest

The authors declare no conflict of interest.
