# Supplementary material for: Oncofertility information interventions in patients with cancer: A systematic review and meta-analysis
Source: Asia Pac J Oncol Nurs. 2026 Apr 13;13:100954. doi: 10.1016/j.apjon.2026.100954 (PMC13141553; doi:10.1016/j.apjon.2026.100954)
Supplement: Multimedia component 1 [file mmc1.docx]

# Supplementary Table S1. Search strategy

| **Databases** | **Details** |
| --- | --- |
| **Pubmed**  **526 results** | #1（21/11/2025; 5476942 results）  ("Neoplasms"[Mesh]) OR ((((((((Neoplasm*[Title/Abstract]) OR (Neoplasia*[Title/Abstract])) OR (Tumor*[Title/Abstract])) OR (Cancer*[Title/Abstract])) OR (Malignant Neoplasm*[Title/Abstract])) OR (Malignancy[Title/Abstract])) OR (Malignancies[Title/Abstract])) OR (Neoplasm*, Malignant[Title/Abstract]))  AND  #2（21/11/2025; 1637753 results）  ((("Reproduction"[Mesh]) OR ("Fertility"[Mesh])) OR ("Fertility Preservation"[Mesh])) OR (((((((((((((((((((((((Fertility[Title/Abstract]) OR (Fecundability[Title/Abstract])) OR (Fecundity[Title/Abstract])) OR (Fertility Incentives[Title/Abstract])) OR (Fertility Incentive[Title/Abstract])) OR (Marital Fertility[Title/Abstract])) OR (Fertility, Marital[Title/Abstract])) OR (Natural Fertility[Title/Abstract])) OR (Fertility, Natural[Title/Abstract])) OR (Fertility Determinant*[Title/Abstract])) OR (Determinant*, Fertility[Title/Abstract])) OR (Fertility Preference*[Title/Abstract])) OR (Preference*, Fertility[Title/Abstract])) OR (Reproducti*[Title/Abstract])) OR (Reproductive Period*[Title/Abstract])) OR (Period*, Reproductive[Title/Abstract])) OR (Fertility Preservation*[Title/Abstract])) OR (Preservation, Fertility[Title/Abstract])) OR (Egg Freezing[Title/Abstract])) OR (Freezing, Egg[Title/Abstract])) OR (Sperm Freezing[Title/Abstract])) OR (Freezing, Sperm[Title/Abstract])) OR (Oncofertility[Title/Abstract]))  AND  #3（21/11/2025; 2772430 results）  ((((((("Counseling"[Mesh]) OR ("Directive Counseling"[Mesh])) OR ("Decision Making"[Mesh])) OR ("Decision Support Techniques"[Mesh])) OR ("Psychotherapy"[Mesh])) OR ("Psychosocial Intervention"[Mesh])) OR ("Interview, Psychological"[Mesh])) OR (((((((((((((((((((((((((((((((((((((Counseling[Title/Abstract]) OR (Directive Counseling[Title/Abstract])) OR (Decision Making[Title/Abstract])) OR (Decision Support Techniques[Title/Abstract])) OR (Psychotherapy[Title/Abstract])) OR (Psychosocial Intervention[Title/Abstract])) OR (interview, psychological[Title/Abstract])) OR (Counseling, Directive[Title/Abstract])) OR (Prescriptive Counseling[Title/Abstract])) OR (Counseling, Prescriptive[Title/Abstract])) OR (Decision Support Techni*[Title/Abstract])) OR (Techni*, Decision Support[Title/Abstract])) OR (Decision Analys*[Title/Abstract])) OR (Analys*, Decision[Title/Abstract])) OR (Decision Aid*[Title/Abstract])) OR (Aid, Decision[Title/Abstract])) OR (Decision Modeling[Title/Abstract])) OR (Modeling, Decision[Title/Abstract])) OR (Model*, Decision Support[Title/Abstract])) OR (Decision Support Model*[Title/Abstract])) OR (Decision Support[Title/Abstract])) OR (Intervention*, Psychosocial[Title/Abstract])) OR (Psychological Intervention*[Title/Abstract])) OR (Intervention*, Psychological[Title/Abstract])) OR (Psychosocial Interventions[Title/Abstract])) OR (Interviews, Psychologic*[Title/Abstract])) OR (Interview, Psychologic[Title/Abstract])) OR (Psychological Interview*[Title/Abstract])) OR (Psychologic Interview*[Title/Abstract])) OR (Aids, Decision[Title/Abstract])) OR (Consultation[Title/Abstract])) OR (Information[Title/Abstract])) OR (Message[Title/Abstract])) OR (Advice[Title/Abstract])) OR (psychoeducation*[Title/Abstract])) OR (psycho*,education*[Title/Abstract])) OR (psychological treatment[Title/Abstract]))  AND  #4（21/11/2025; 1919277 results）  (("Clinical Trials as Topic"[Mesh:NoExp]) OR ((((((((((((randomized controlled trial[Publication Type]) OR (controlled clinical trial[Publication Type])) OR (clinical trial[Publication Type])) OR (RCT[Title/Abstract])) OR (random allocation[Title/Abstract])) OR (Randomization[Title/Abstract])) OR (randomized[Title/Abstract])) OR (randomised[Title/Abstract])) OR (placebo[Title/Abstract])) OR (randomly[Title/Abstract])) OR (randomized experiment[Title/Abstract])) OR (trial[Title])) NOT ("animals"[Mesh] NOT "humans"[Mesh])) |
| **Web of Science**  **(Core Collection）**  **635 results** | #1（21/11/2025; 4318142 results）  TS=(Neoplasm* OR Neoplasia* OR Tumor* OR Cancer* OR Malignant Neoplasm* OR Malignancy OR Malignancies OR Neoplasm*,  Malignant)  AND  #2（21/11/2025; 670991 results）  TS=(Fertility OR Fecundability OR Fecundity OR Fertility Incentives OR Fertility Incentive OR Marital Fertility OR Fertility, Marital OR Natural Fertility OR Fertility, Natural OR Fertility Determinant* OR Determinant*, Fertility OR Fertility Preference* OR Preference*, Fertility OR Reproducti* OR Reproductive Period* OR Period*, Reproductive OR Fertility Preservation* OR Preservation, Fertility OR Egg Freezing OR Freezing, Egg OR Sperm Freezing OR Freezing, Sperm OR Oncofertility)  AND  #3（21/11/2025; 5670855 results）  TS=(Counseling OR Directive Counseling OR Decision Making OR Decision Support Techniques OR Psychotherapy OR Psychosocial Intervention OR interview, psychological OR Counseling, Directive OR Prescriptive Counseling OR Counseling, Prescriptive OR Decision Support Techni* OR Techni*, Decision Support OR Decision Analys* OR Analys*, Decision OR Decision Aid* OR Aid, Decision OR Decision Modeling OR Modeling, Decision OR Model*, Decision Support OR Decision Support Model* OR Decision Support OR Intervention*, Psychosocial OR Psychological Intervention* OR Intervention*, Psychological OR Psychosocial Interventions OR Interviews, Psychologic* OR Interview, Psychologic OR Psychological Interview* OR Psychologic Interview* OR Aids, Decision OR Consultation OR Information OR Message OR Advice OR psychoeducation* OR psycho*,education* OR psychological treatment)  AND  #4（21/11/2025; 2816881 results）  TS=(Clinical Trial* OR randomi?ed controlled trial OR controlled clinical trial OR clinical trial OR rct OR random allocation OR randomly allocated OR allocated randomly OR Randomization OR randomi?ed OR placebo* OR randomly OR randomized experiment OR trial) |
| **Scopus**  **1108 results** | #1（21/11/2025; 6797722 results）  TITLE-ABS-KEY ( neoplasm* OR neoplasia* OR tumor* OR cancer* OR "Malignant Neoplasm*" OR malignancy OR malignancies OR "Neoplasm*, Malignant" )  AND  #2（21/11/2025; 1104118 results）  TITLE-ABS-KEY ( fertility OR fecundability OR fecundity OR "Fertility Incentives" OR "Fertility Incentive" OR "Marital Fertility" OR "Fertility, Marital" OR "Natural Fertility" OR "Fertility, Natural" OR "Fertility Determinant*" OR "Determinant*, Fertility" OR "Fertility Preference*" OR "Preference*, Fertility" OR reproducti* OR "Reproductive Period*" OR "Period*, Reproductive" OR "Fertility Preservation*" OR "Preservation, Fertility" OR "Egg Freezing" OR "Freezing, Egg" OR "Sperm Freezing" OR "Freezing, Sperm" OR oncofertility )  AND  #3（21/11/2025; 9536094 results）  TITLE-ABS-KEY ( counseling OR "Directive Counseling" OR "Decision Making" OR "Decision Support Techniques" OR psychotherapy OR "Psychosocial Intervention" OR "interview, psychological" OR "Counseling, Directive" OR "Prescriptive Counseling" OR "Counseling, Prescriptive" OR "Decision Support Techni*" OR "Techni*, Decision Support" OR "Decision Analys*" OR "Analys*, Decision" OR "Decision Aid*" OR "Aid, Decision" OR "Decision Modeling" OR "Modeling, Decision" OR "Model*, Decision Support" OR "Decision Support Model*" OR "Decision Support" OR "Intervention*, Psychosocial" OR "Psychological Intervention*" OR "Intervention*, Psychological" OR "Psychosocial Interventions" OR "Interviews, Psychologic*" OR "Interview, Psychologic" OR "Psychological Interview*" OR "Psychologic Interview*" OR "Aids, Decision" OR consultation OR information OR message OR advice OR psychoeducation* OR "psycho*,education*" OR "psychological treatment" )  AND  #4（21/11/2025; 3597599 results）  TITLE-ABS-KEY ( "randomi?ed controlled trial" OR "controlled clinical trial" OR "clinical trial" OR randomized OR randomised OR placebo OR randomly ) OR TITLE ( trial ) |
| **Embase**  **1077 results** | #1（21/11/2025; 7120772 results）  ('malignant neoplasm'/exp OR (cancer*:ab,ti OR 'malignant neoplasia':ab,ti OR 'malignant neoplastic disease':ab,ti OR 'malignant tumor':ab,ti OR 'malignant tumour':ab,ti OR 'neoplasia, malignant':ab,ti OR 'neoplasmic malignancy':ab,ti OR 'neoplastic malignancy':ab,ti OR 'oncologic* malignancy':ab,ti OR 'tumor, malignant':ab,ti OR 'tumor* malignancy':ab,ti OR 'tumour, malignant':ab,ti OR 'malignant neoplasm':ab,ti OR tumor*:ab,ti OR tumour*:ab,ti))  AND  #2（21/11/2025; 2010559 results）  (('fertility'/exp OR 'reproduction'/exp OR 'fertility preservation'/exp) OR (fecundity:ab,ti OR 'sperm-ovum interactions':ab,ti OR fertility:ab,ti OR 'human reproduction':ab,ti OR 'reproductive function':ab,ti OR reproductive:ab,ti OR 'sexual reproduction':ab,ti OR reproduction:ab,ti OR 'fertility preservation':ab,ti))  AND  #3（21/11/2025; 1403822 results）  (('counseling'/exp OR 'directive counseling'/exp OR 'decision making'/exp OR 'decision support system'/exp OR 'psychotherapy'/exp OR 'psychosocial intervention'/exp OR 'psychological interview'/exp) OR (counselling:ab,ti OR counseling:ab,ti OR 'directive counseling':ab,ti OR 'choice behavior':ab,ti OR 'choice behaviour':ab,ti OR 'choice making':ab,ti OR 'decision process':ab,ti OR decisionmaking:ab,ti OR judgement:ab,ti OR judgment:ab,ti OR 'decision making':ab,ti OR 'decision making, computer-assisted':ab,ti OR 'decision support':ab,ti OR 'decision support techniques':ab,ti OR 'decision support system':ab,ti OR 'holistic psychotherapy':ab,ti OR 'multiple psychotherapy':ab,ti OR 'psychotherapeutic training':ab,ti OR 'psychotherapy, multiple':ab,ti OR 'socioenvironmental therapy':ab,ti OR psychotherapy:ab,ti OR 'psycho-social intervention':ab,ti OR 'psycho-social therapy':ab,ti OR 'psycho-social treatment':ab,ti OR 'psychosocial therapy':ab,ti OR 'psychosocial treatment':ab,ti OR 'psychosocial intervention':ab,ti OR 'interview, psychological':ab,ti OR 'psychological interview':ab,ti))  AND  #4（21/11/2025; 3836530 results）  ('clinical trial'/de OR 'randomized controlled trial'/de OR 'randomization'/de OR 'single blind procedure'/de OR 'double blind procedure'/de OR 'crossover procedure'/de OR 'placebo'/de OR 'prospective study'/de OR ('randomi?ed controlled' NEXT/1 trial*) OR rct OR 'randomly allocated' OR 'allocated randomly' OR 'random allocation' OR (allocated NEAR/2 random) OR (single NEXT/1 blind*) OR (double NEXT/1 blind*) OR ((treble OR triple) NEAR/1 blind*) OR placebo*) |
| **Cochrane Library**  **477 results** | #1 MeSH descriptor: [Neoplasms] explode all trees  #2 (Neoplasm*):ti,ab,kw OR (Neoplasia*):ti,ab,kw OR (Tumor*):ti,ab,kw OR (Cancer*):ti,ab,kw OR ("Malignant Neoplasm"):ti,ab,kw OR (Malignancy):ti,ab,kw OR (Malignancies):ti,ab,kw OR ("Malignant Neoplasms"):ti,ab,kw  #3 #1 OR #2  #4 MeSH descriptor: [Fertility] explode all trees  #5 MeSH descriptor: [Fertility Preservation] explode all trees  #6 MeSH descriptor: [Reproduction] explode all trees  #7 (Fertility):ti,ab,kw OR (Fecundability):ti,ab,kw OR (Fecundity):ti,ab,kw OR ("Fertility Incentives"):ti,ab,kw OR ("Fertility Incentive"):ti,ab,kw OR ("Marital Fertility"):ti,ab,kw OR ("Fertility, Marital"):ti,ab,kw OR ("Natural Fertility"):ti,ab,kw OR ("Fertility, Natural"):ti,ab,kw OR ("Fertility Determinant"):ti,ab,kw OR ("Fertility Determinants"):ti,ab,kw OR ("Fertility Preferences"):ti,ab,kw OR ("Fertility Preference"):ti,ab,kw OR (Reproducti*):ti,ab,kw OR ("Reproductive Periods"):ti,ab,kw OR ("Reproductive Period"):ti,ab,kw OR ("Fertility Preservation"):ti,ab,kw OR ("Egg Freezing"):ti,ab,kw OR ("Freezing, Egg"):ti,ab,kw OR ("Sperm Freezing"):ti,ab,kw OR ("Freezing, Sperm"):ti,ab,kw OR (Oncofertility):ti,ab,kw  #8 #4 OR #5 OR #6 OR #7  #9 MeSH descriptor: [Decision Making] explode all trees  #10 MeSH descriptor: [Decision Support Techniques] explode all trees  #11 MeSH descriptor: [Counseling] explode all trees  #12 MeSH descriptor: [Directive Counseling] explode all trees  #13 MeSH descriptor: [Psychotherapy] explode all trees  #14 MeSH descriptor: [Interview, Psychological] explode all trees  #15 MeSH descriptor: [Psychosocial Intervention] explode all trees  #16 (Counseling):ti,ab,kw OR ("Directive Counseling"):ti,ab,kw OR ("Decision Making"):ti,ab,kw OR ("Decision Support Techniques"):ti,ab,kw OR (Psychotherapy):ti,ab,kw OR ("Psychosocial Intervention"):ti,ab,kw OR ("interview, psychological"):ti,ab,kw OR ("Counseling, Directive"):ti,ab,kw OR ("Prescriptive Counseling"):ti,ab,kw OR ("Counseling, Prescriptive"):ti,ab,kw OR ("Decision Support Technics"):ti,ab,kw OR ("Decision Support Technic"):ti,ab,kw OR ("Decision Analysis"):ti,ab,kw OR ("Decision Analyses"):ti,ab,kw OR ("Decision Aid"):ti,ab,kw OR ("Decision Aids"):ti,ab,kw OR ("Decision Modeling"):ti,ab,kw OR ("Modeling, Decision"):ti,ab,kw OR ("Decision Support Models"):ti,ab,kw OR ("Decision Support Model"):ti,ab,kw OR ("Decision Support"):ti,ab,kw OR ("Psychological Interventions"):ti,ab,kw OR ("Psychological Intervention"):ti,ab,kw OR ("Psychosocial Intervention"):ti,ab,kw OR ("Psychosocial Interventions"):ti,ab,kw OR ("Psychologic Interviews"):ti,ab,kw OR ("Psychologic Interview"):ti,ab,kw OR ("Psychological Interview"):ti,ab,kw OR ("Psychological Interviews"):ti,ab,kw OR ("Aids, Decision"):ti,ab,kw OR (Consultation):ti,ab,kw OR (Information):ti,ab,kw OR (Message):ti,ab,kw OR (Advice):ti,ab,kw OR (psychoeducation*):ti,ab,kw OR ("psychological education"):ti,ab,kw OR ("psychological treatment"):ti,ab,kw OR (psycho-educational):ti,ab,kw OR ("psychologic education"):ti,ab,kw  #17 #9 OR #10 OR #11 OR #12 OR #13 OR #14 OR #15 OR #16  #18 #3 AND #8 AND #17  in Trials |

# Supplementary Table S2. Detailed risk of bias assessment in RoB 2

| **Study** | **Randomization** | **Deviations from intended intervention** | **Missing data** | **Outcome measurement** | **Selection of reported result** | **Overall** |
| --- | --- | --- | --- | --- | --- | --- |
| Barjasteh et al.(2022) | Low | Low | Low | Some concerns | Some concerns | Some concerns |
| Canada et al.(2007) | Some concerns | Some concerns | Some concerns | Some concerns | Some concerns | Some concerns |
| Dong et al.(2024) | Low | Some concerns | Low | Some concerns | Some concerns | Some concerns |
| Ehrbar et al.(2021) | Some concerns | High | High | Low | Low | High |
| Ehrbar et al.(2019) | Some concerns | High | High | Low | Low | High |
| Huang et al.(2022) | Some concerns | Some concerns | Low | Some concerns | Some concerns | Some concerns |
| Huyghe et al.(2009) | Some concerns | Low | Low | Some concerns | Some concerns | Some concerns |
| Koizumi et al.(2023) | Low | Low | Low | Low | Low | Low |
| Micaux et al.(2022) | Low | Low | Low | Some concerns | Some concerns | Some concerns |
| Partridge et al.(2019) | Low | Low | Low | Low | Low | Low |
| Nahata et al.(2025) | Low | Some concerns | Low | Low | Some concerns | Some concerns |
