# Supplementary material for: Oncofertility information interventions in patients with cancer: A systematic review and meta-analysis
Source: Asia Pac J Oncol Nurs. 2026 Apr 13;13:100954. doi: 10.1016/j.apjon.2026.100954 (PMC13141553; doi:10.1016/j.apjon.2026.100954)

# Supplementary Fig. S1. The subgroup analysis of the effects of oncofertility information support on fertility-related knowledge

1. Regions


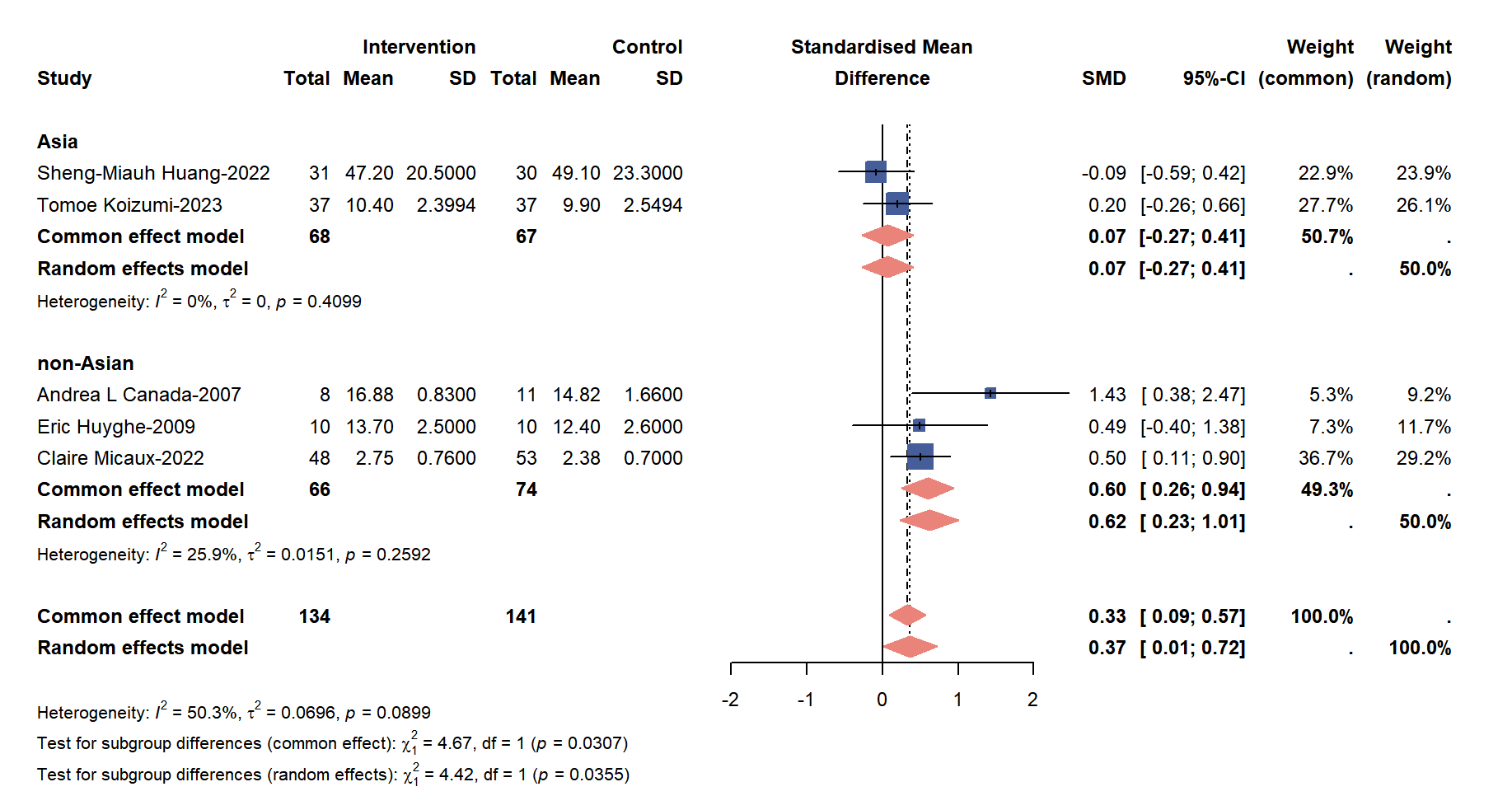


1. Cancer types


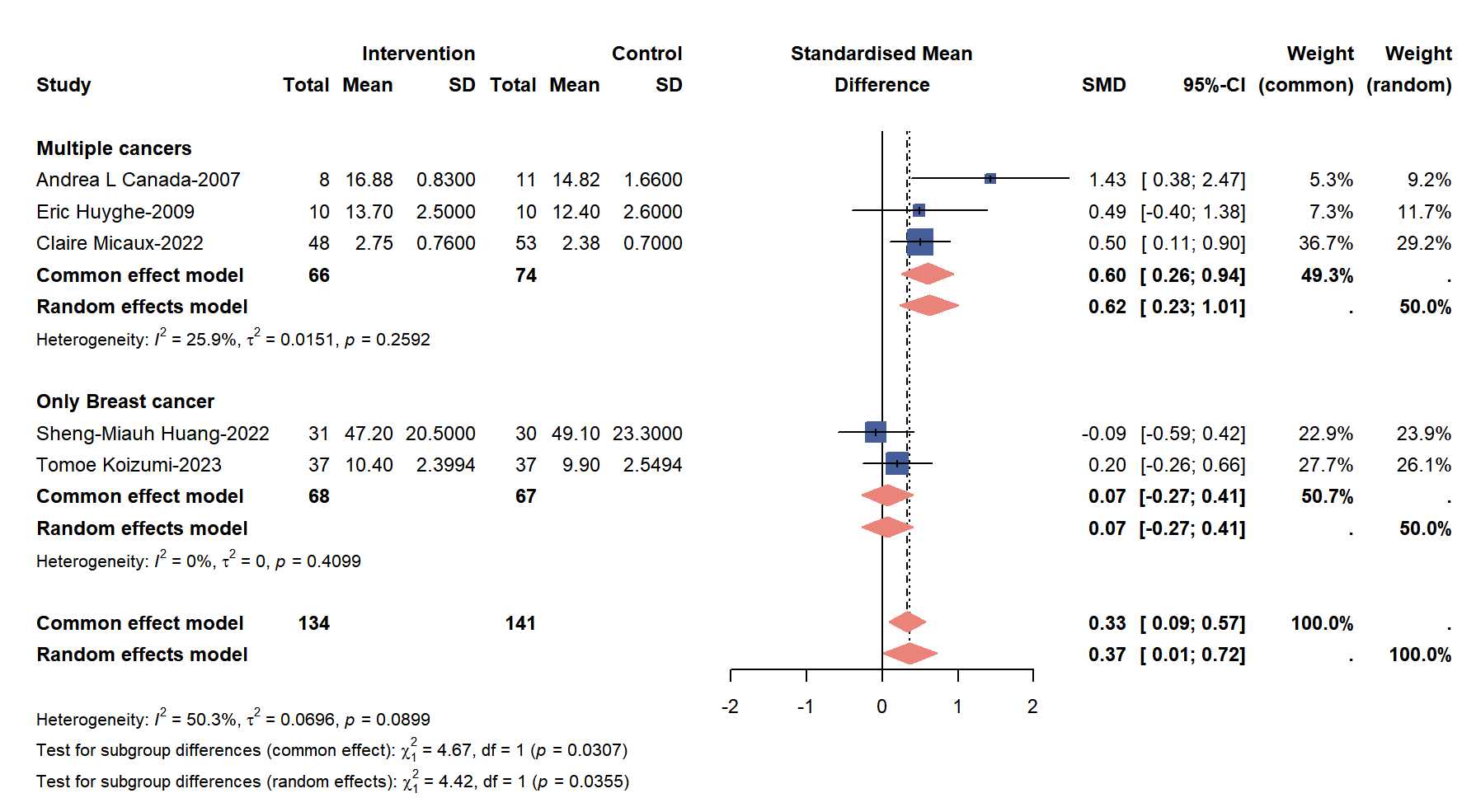


1. Intervention formats


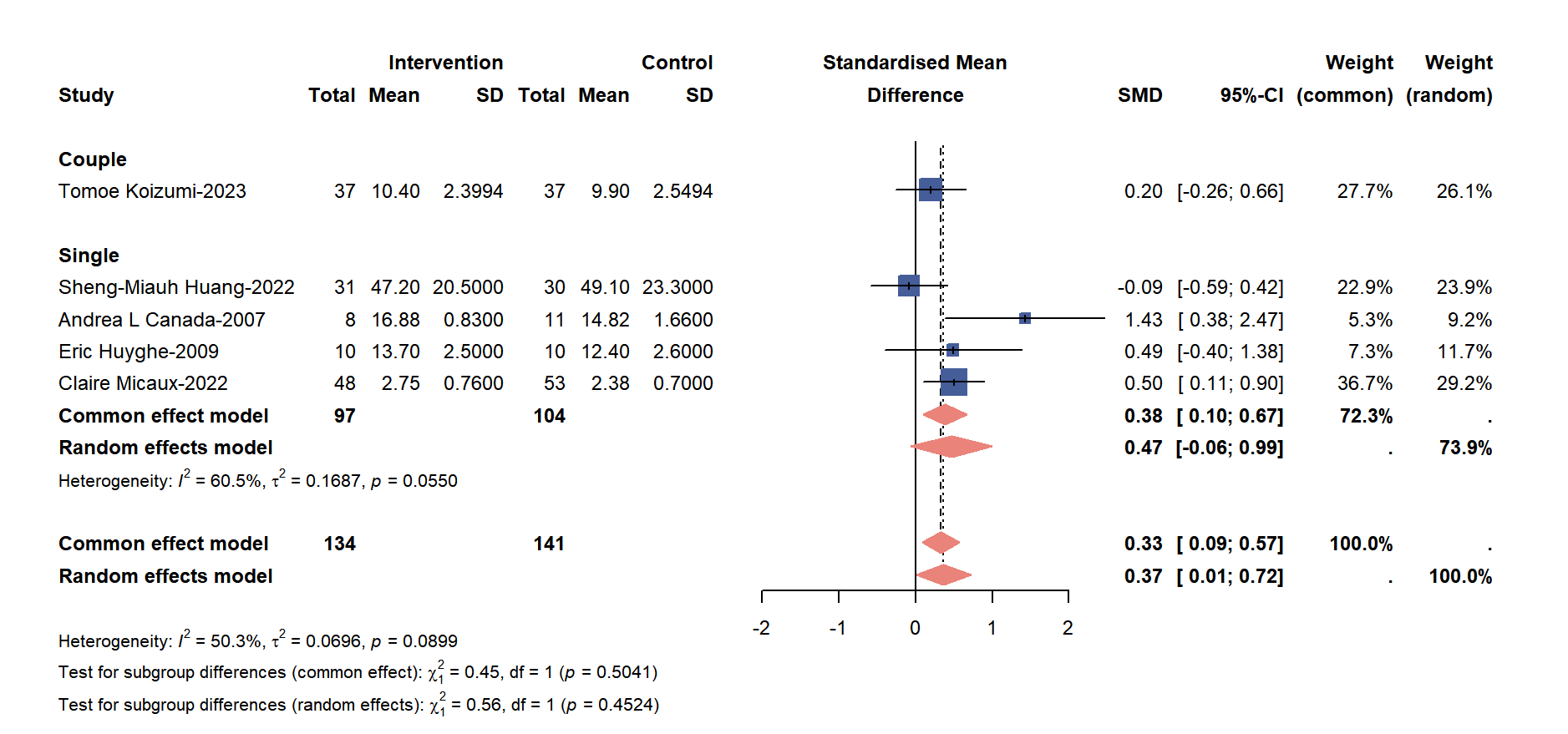


1. Delivery modes


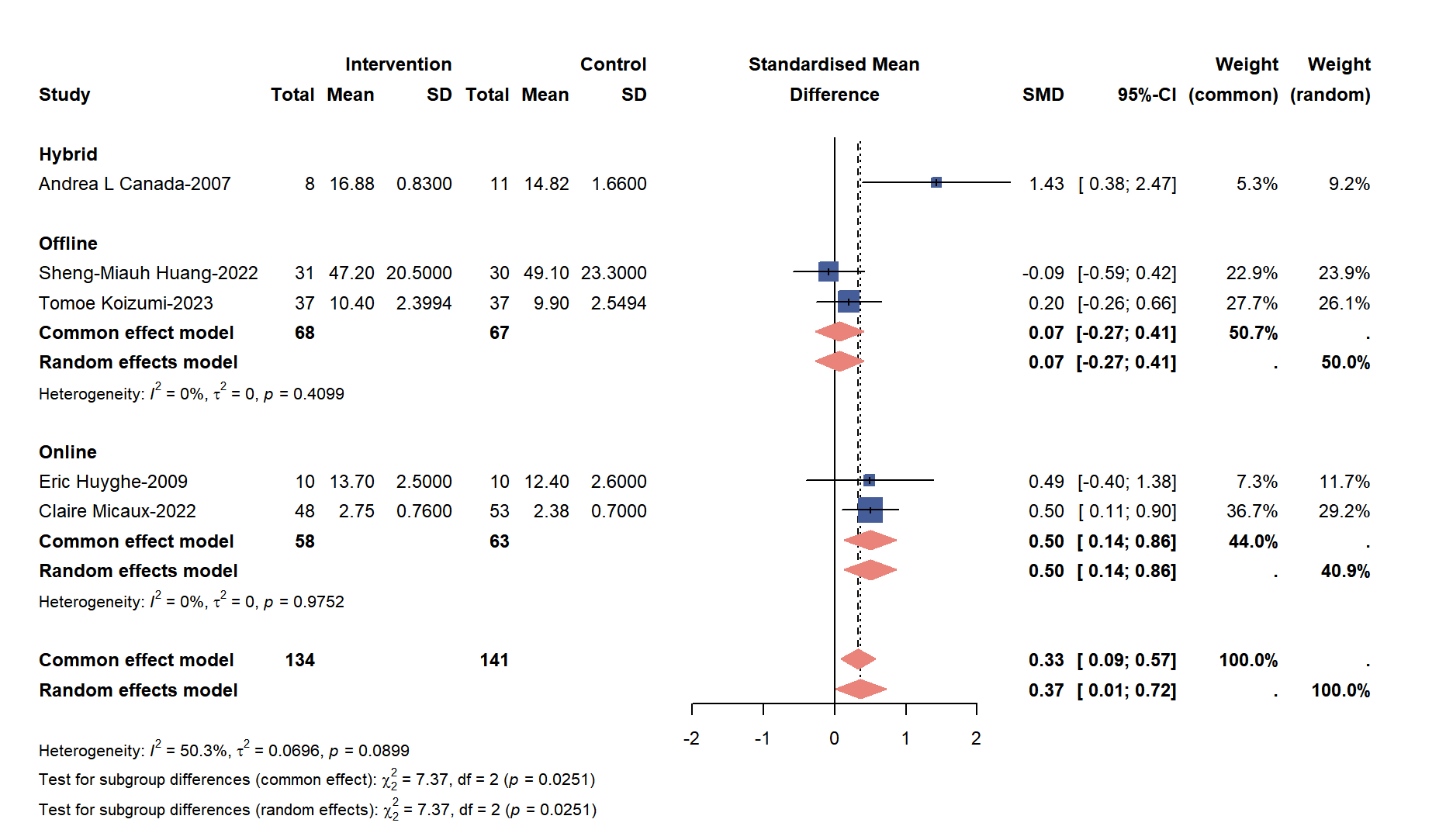


1. Session formats


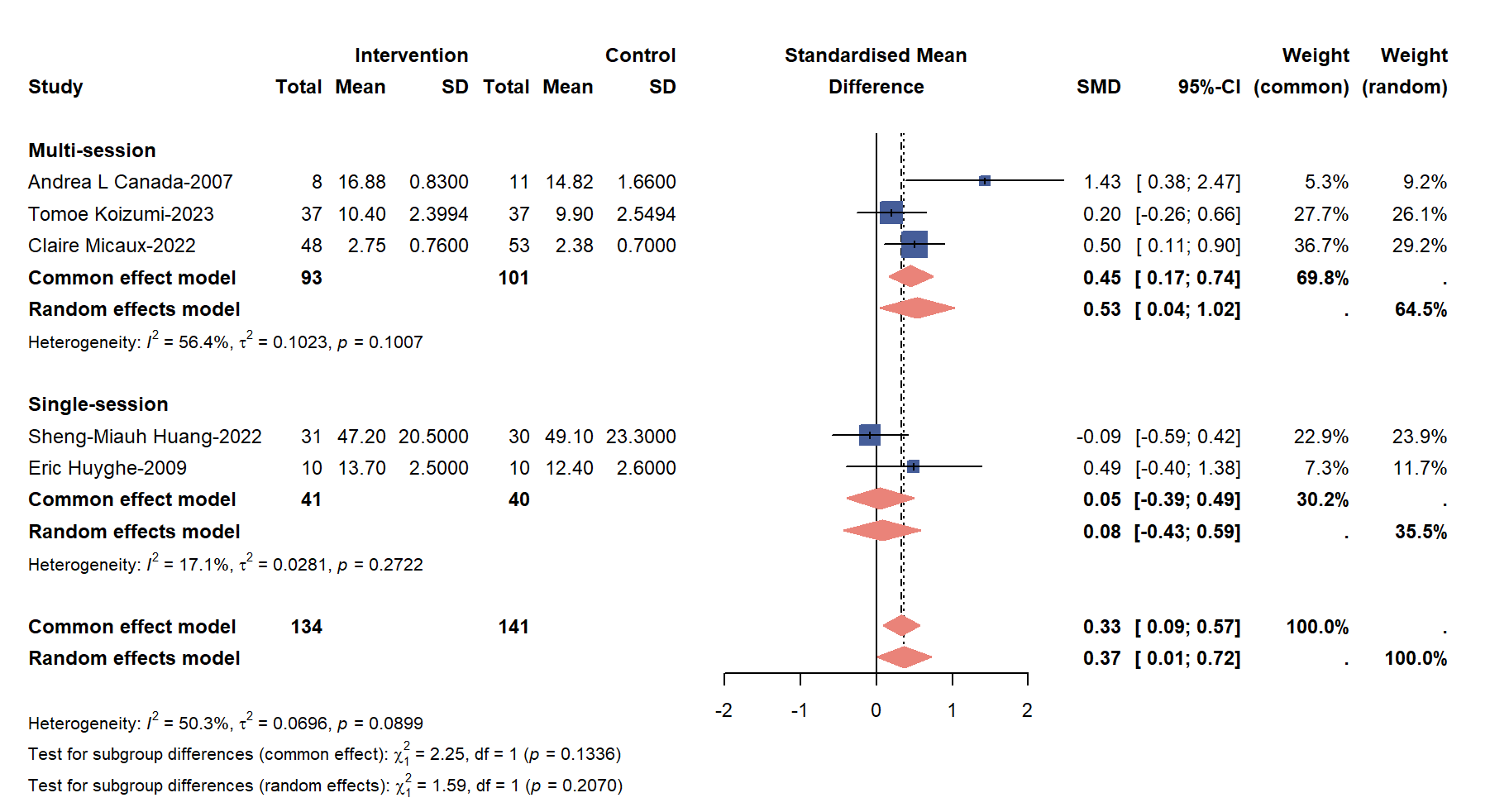


# Supplementary Fig. S2. The forest plot showing the effect of oncofertility information support on other outcomes

1. Quality of life


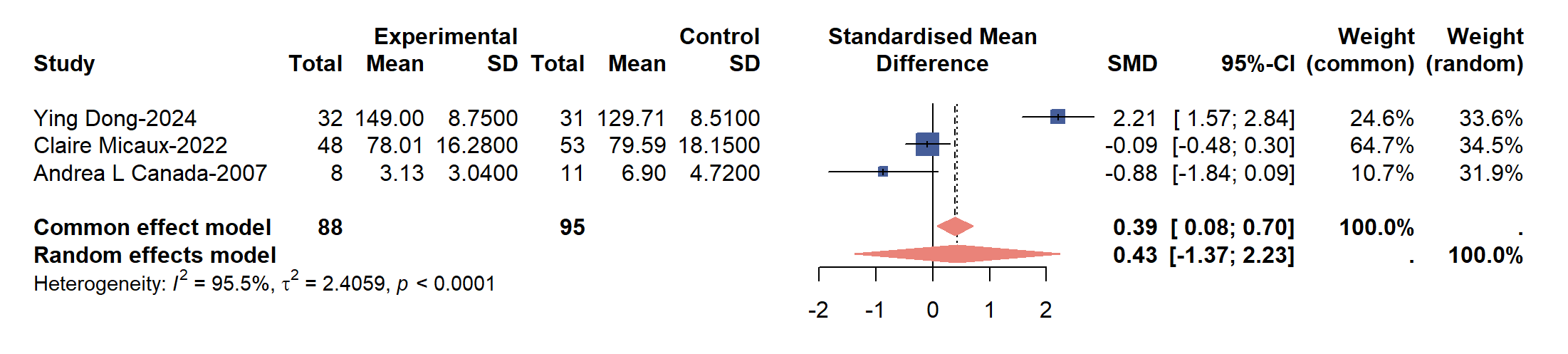


1. Anxiety and depression


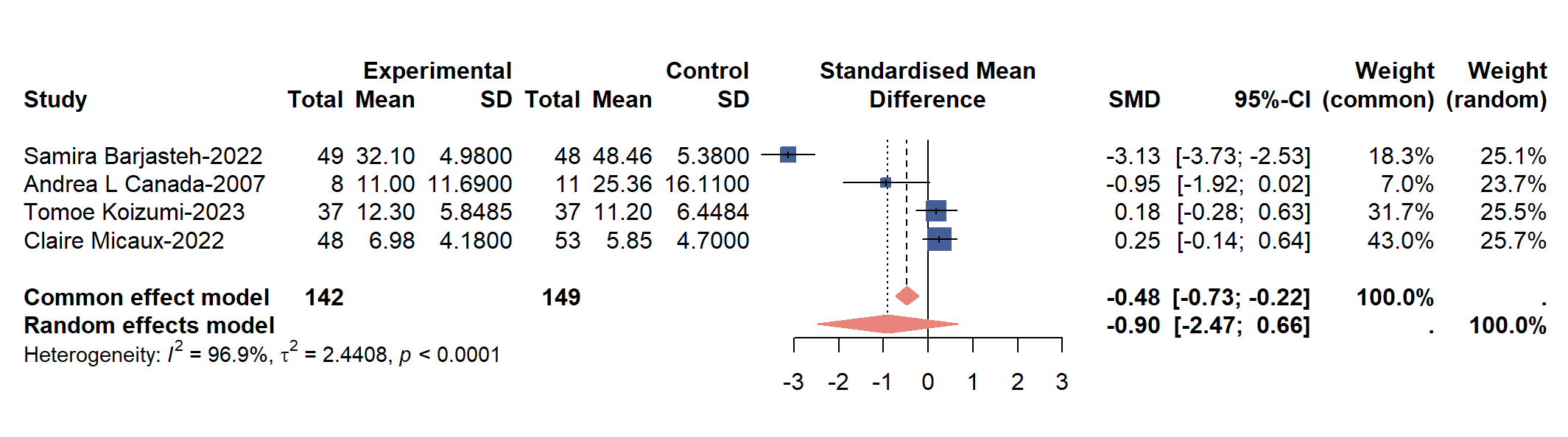


1. Fertility intentions


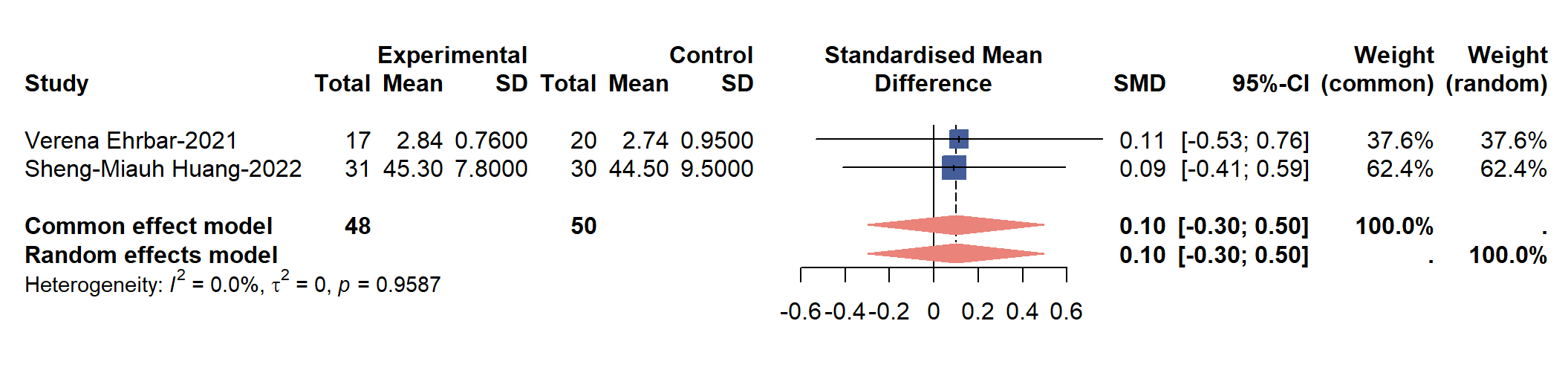


# Supplementary Fig. S3. Sensitivity analysis

1. Fertility-related knowledge


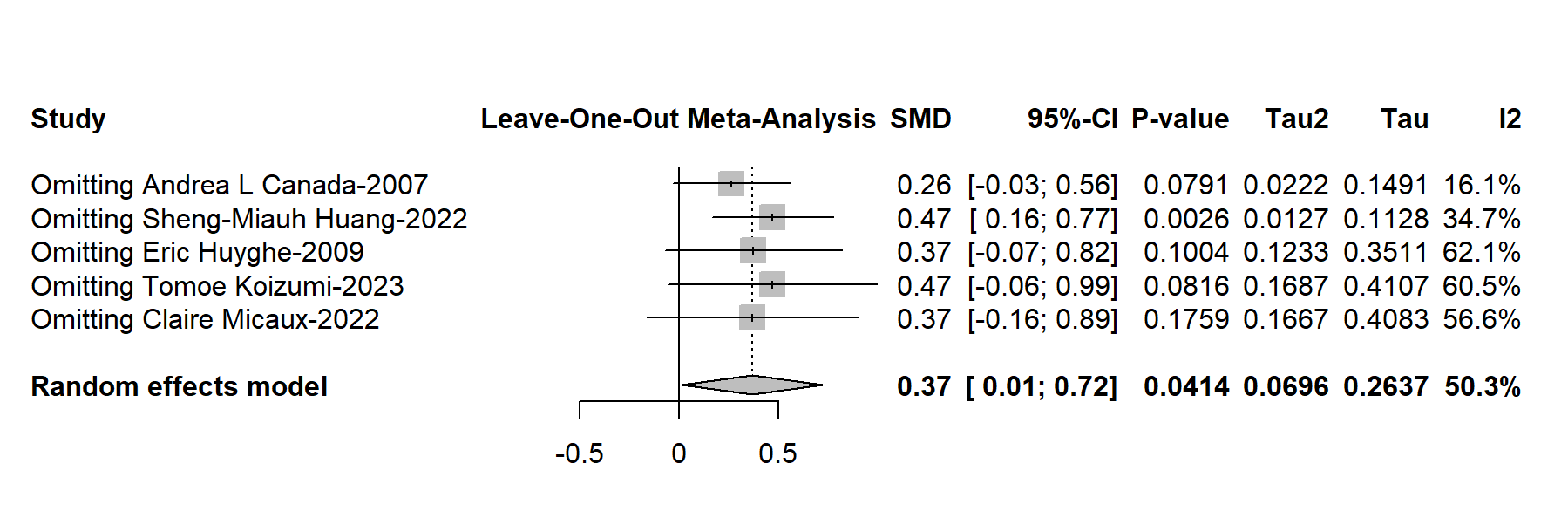


1. Reproductive concerns


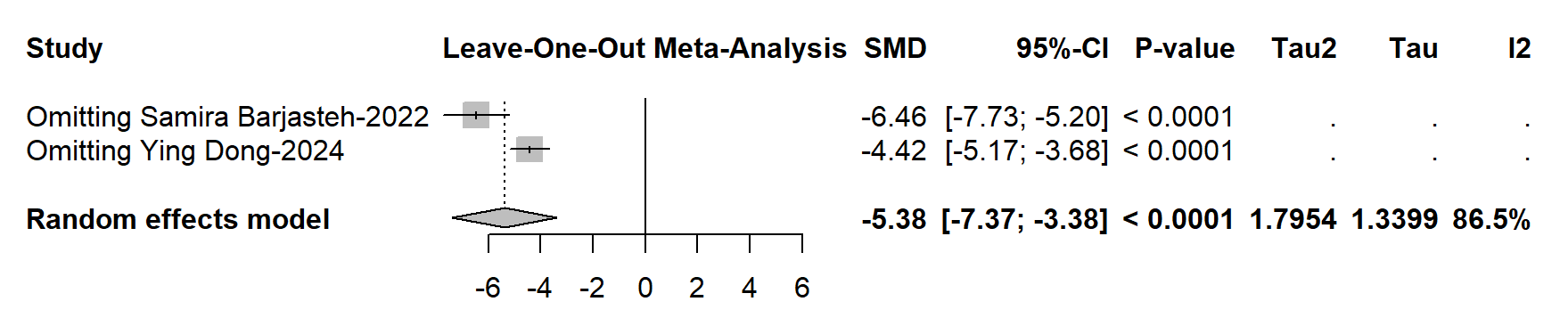


1. Decision conflict


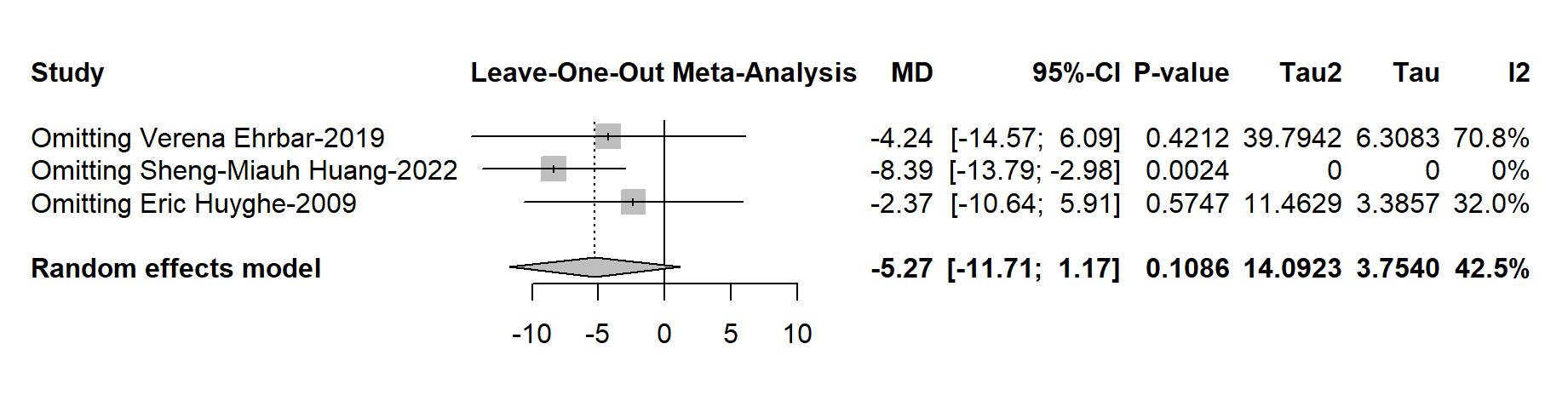


1. Decisional regret


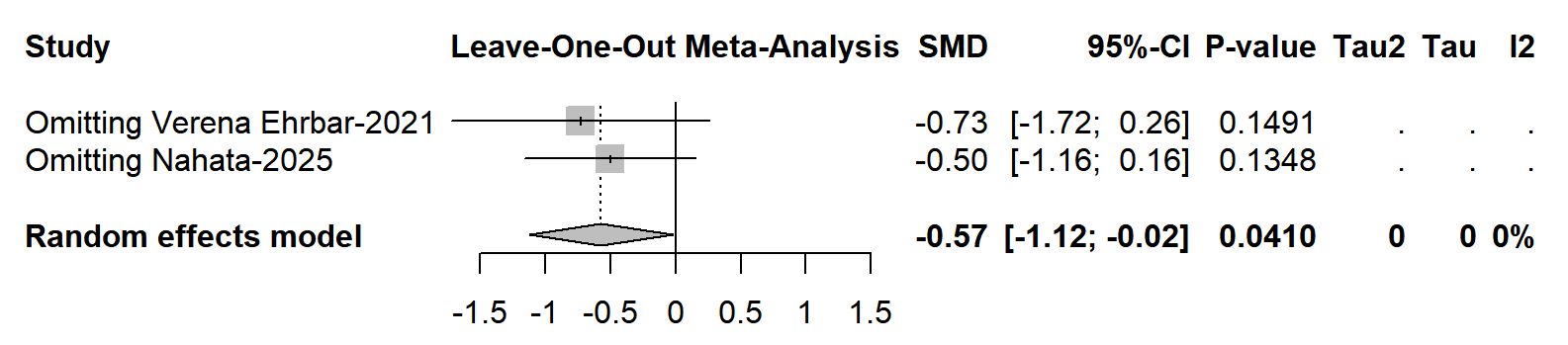


1. Quality of life


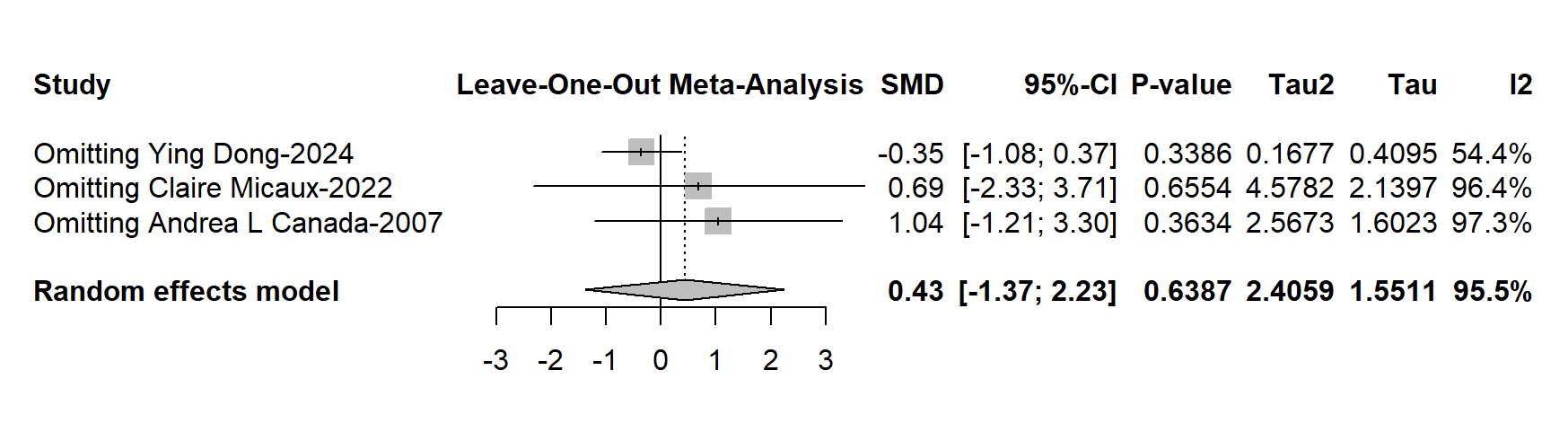


1. Anxiety and depression


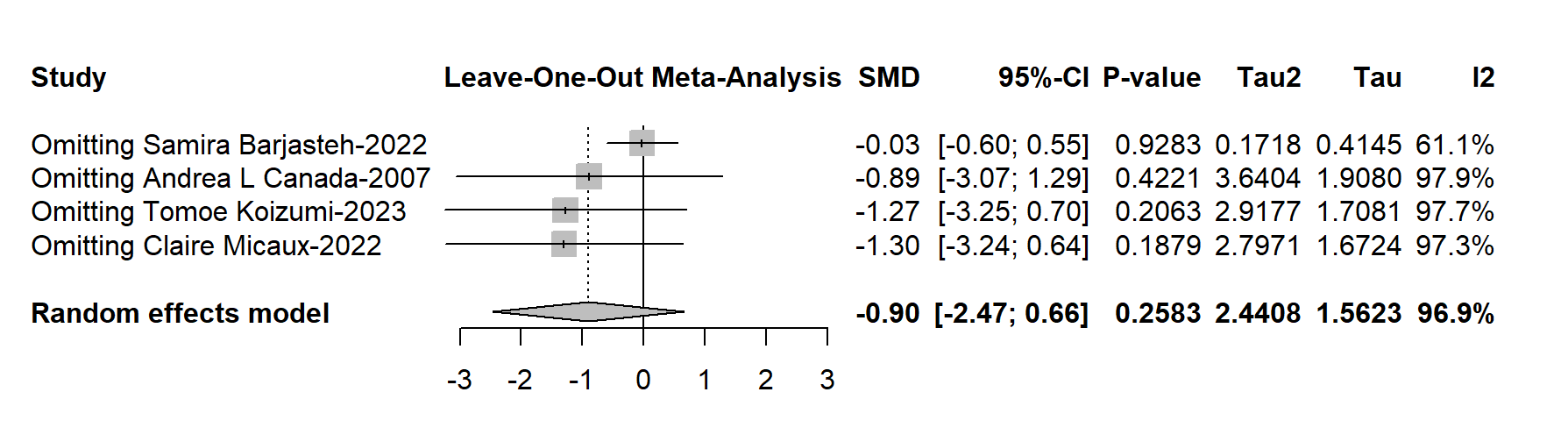


1. Fertility intentions


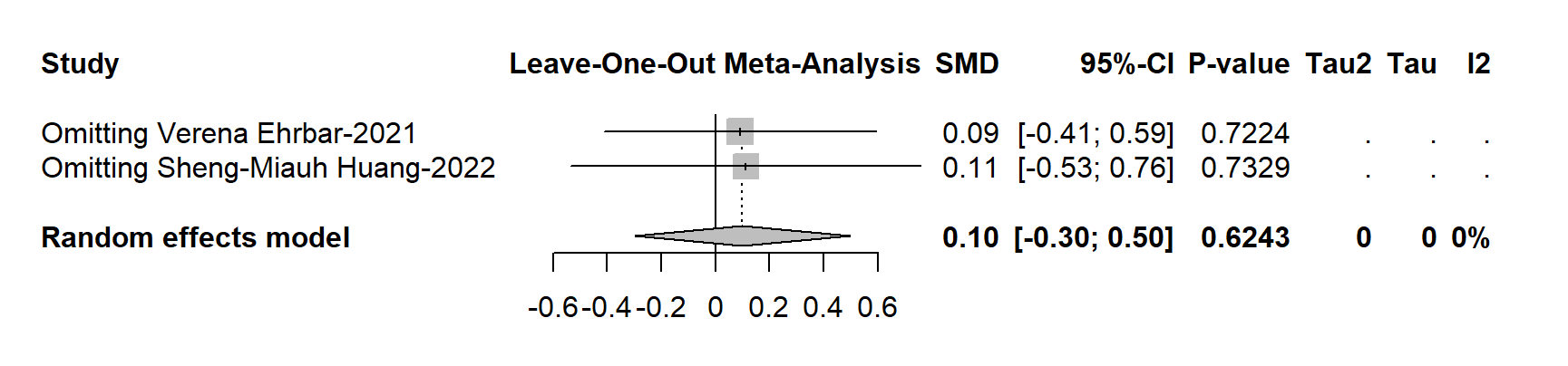


# Supplementary Fig. S4. Certainty of evidence assessed using the GRADE system.


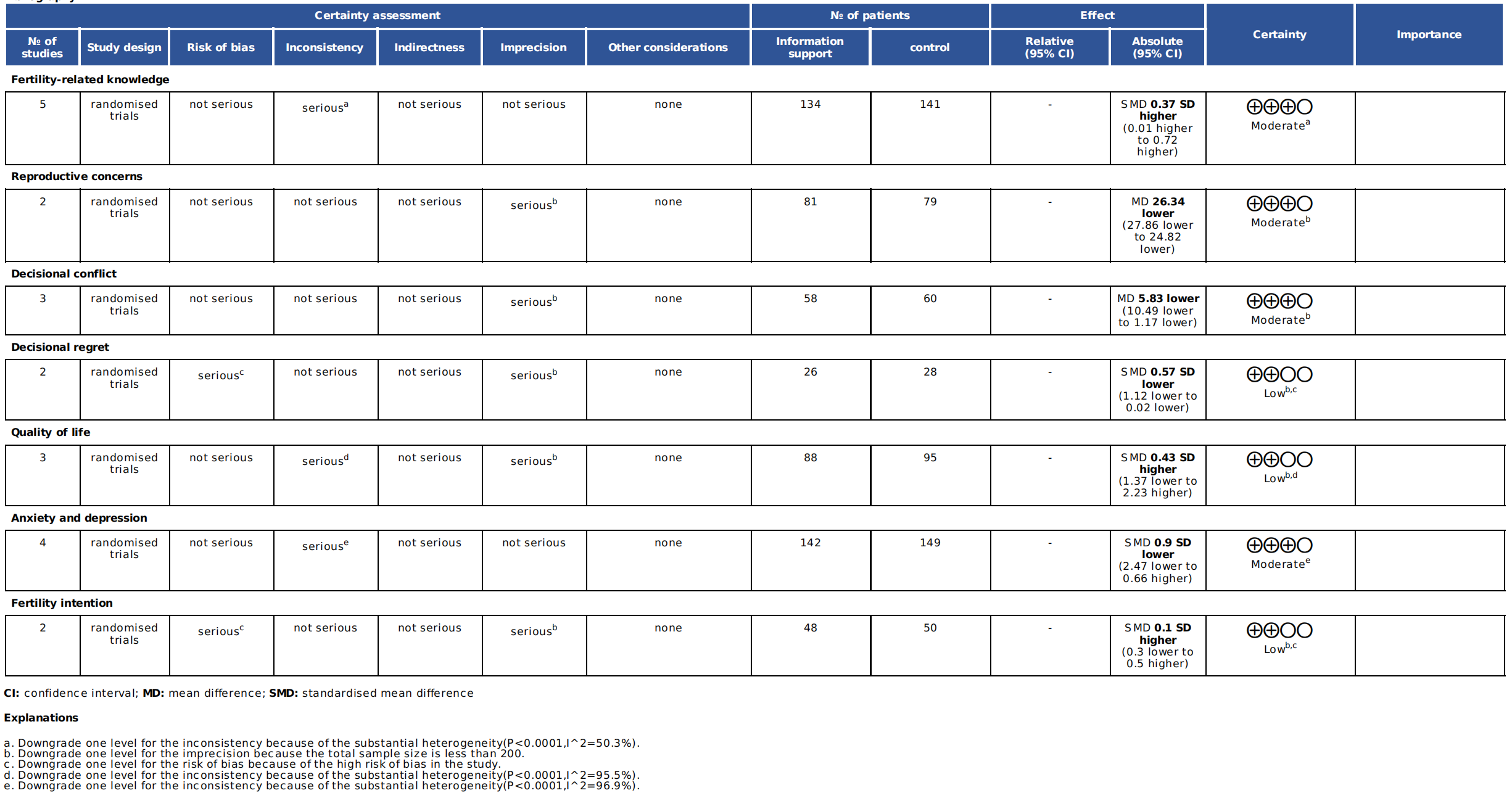

Supplement: Multimedia component 2 [file mmc2.docx]
